# Physiological and interactomic analysis reveals versatile functions of *Arabidopsis* 14-3-3 quadruple mutants in response to Fe deficiency

**DOI:** 10.1038/s41598-021-94908-9

**Published:** 2021-07-30

**Authors:** Jing Gao, Paula J. M. van Kleeff, Ka Wan Li, Albertus H. de Boer

**Affiliations:** 1 Institute of Apicultural Research, Chinese Academy of Agricultural Sciences, Beijing, 100093 China; 2 Department of Structural Biology, Faculty of Earth and Life Sciences, Vrije Universiteit Amsterdam, De Boelelaan 1085, 1081 HV Amsterdam, the Netherlands; 3 Department of Molecular and Cellular Neurobiology, Faculty of Earth and Life Sciences, Center for Neurogenomics and Cognitive Research, Vrije Universiteit Amsterdam, De Boelelaan 1085, 1081 HV Amsterdam, The Netherlands; 4 Department of Plant Physiology, Swammerdam Institute for Life Sciences, University of Amsterdam, Kruislaan 318, 1098 SM Amsterdam, The Netherlands; 5 Department of Medicinal Chemistry, Beta Faculty, Vrije Universiteit Amsterdam, De Boelelaan 1108, 1081 HZ Amsterdam, The Netherlands

**Keywords:** Plant physiology, Plant stress responses

## Abstract

To date, few phenotypes have been described for *Arabidopsis* 14-3-3 mutants or the phenotypes showing the role of 14-3-3 in plant responding to abiotic stress. Although one member of the 14-3-3 protein family (14-3-3 *omicron*) was shown to be involved in the proper operation of Fe acquisition mechanisms at physiological and gene expression levels in *Arabidopsis thaliana*, it remains to be explored whether other members play a role in regulating iron acquisition. To more directly and effectively observe whether members of 14-3-3 non-epsilon group have a function in Fe-deficiency adaptation, three higher order quadruple KOs, kappa/lambda/phi/chi (*klpc*), kappa/lambda/upsilon/nu(*klu*n), and upsilon/nu/phi/chi (*unpc*) were generated and studied for physiological analysis in this study. The analysis of iron-utilization efficiency, root phenotyping, and transcriptional level of Fe-responsive genes suggested that the mutant with *kl* background showed different phenotypes from Wt when plants suffered Fe starved, while these phenotypes were absent in the *unpc* mutant. Moreover, the absence of the four 14-3-3 isoforms in the *klun* mutant has a clear impact on the 14-3-3 interactome upon Fe deficiency. Dynamics of 14-3-3-client interactions analysis showed that 27 and 17 proteins differentially interacted with 14-3-3 in Wt and *klun* roots caused by Fe deficiency, respectively. Many of these Fe responsive proteins have a role in glycolysis, oxidative phosphorylation and TCA cycle, the FoF1-synthase and in the cysteine/methionine synthesis. A clear explanation for the observed phenotypes awaits a more detailed analysis of the functional aspects of 14-3-3 binding to the target proteins identified in this study.

## Introduction

Iron deficiency may lead to decreases in vegetative growth and quality losses, also known as "lime-induced chlorosis". Although iron is the fourth most abundant element in the earth’s crust, it is mainly in the Fe^3+^ form and not soluble in soils. So far, plants have developed two distinct strategies to enhance the uptake of the poorly soluble iron compounds from soil: i) the reduction strategy (strategy I) employed by non-graminaceous plants, involving in acidification of the rhizosphere followed by Fe^3+^ reduction to Fe^2+^ prior to iron uptake, and ii) the chelation strategy (strategy II) employed by graminaceous plants, relies on the exudation of phytosiderophores, which can rapidly chelate Fe^3+^ and mobilize iron in soil solution^1^. In strategy I plants, such as *Arabidopsis*, FERRIC REDUCTION OXIDASE 2 (FRO2) is responsible for reduction of Fe^3+^ to Fe^2+^ on the root surface, and Fe^2+^ is taken up through the root plasma membrane by IRON-REGULATED TRANSPORTER 1 (IRT1)^2,3^. The basic helix-loop-helix (bHLH) transcription factor FIT, as a central transcriptional factor involved in iron homeostasis, positively regulates the expression of FRO2, IRT1, and AHA2 (H^+^-translocating P- type ATPase)^4,5^. In addition to this root transport system, The citrate transporter FRD3 transports Fe to the xylem sap for distribution of Fe throughout the plant^6^. For this long-distant transport, Fe^3+^ is likely chelated by citrate in the xylem sap. It has been reported that synthesis and excretion of phenolic compounds promote reutilization of root apoplastic Fe^7^. Secreted coumarins through the PLEIOTROPIC DRUG RESISTANCE PROTEIN 9 (PDR9) will enhance Fe (III) availability to the FRO2 to generate Fe^2+^, thereby can be transported into the plant through IRT1, especially in alkaline soil^8^.

14-3-3 proteins are a class of molecular chaperones that bind to and thereby influence the function of phosphorylated proteins. Members of the 14-3-3 protein family, known as important regulators in osmotic stress and salt stress responses in plants, were also found as a key regulators required for the proper operation of Fe acquisition mechanisms at physiological and gene expression levels in *Arabidopsis thaliana*^9^. Loss-of-function of *grf11*(14-3-3 chi) resulted in failure of rhizosphere acidification and ferric chelate reductase (FCR) induction, and thus decreased Fe uptake. Moreover, expression of *IRT1*, *FRO2*, and *AHA2*, were repressed in the *grf11* mutant both under Fe-deficient and under Fe-sufficient conditions. Recently, Singh et al. have demonstrated that a key factor, NON-RESPONSE TO Fe-DEFICIENCY 2 (NRF2)/EARLY FLOWERING 8 (ELF8), controls Fe- deficiency response via GRF11 by the activation of histone H3 lysine 4 trimethylation (H3K4me3) in plant roots^10^. In detergent-resistant membranes (DRMs) of sugar beet roots, the Fe deficiency induced a decrease in abundance of six 14-3-3 like proteins, which would be in line with the decreases in relative abundance of an important number of kinases, e.g. CDPK (calcium dependent protein kinase). In addition, quantitative proteomics^11,12^, as well as a phosphoproteomics profile^13^ of plants exposed to Fe deficiency, show that some putative 14-3-3 targets, such as fructose-bisphosphate aldolase, cytosolic alkaline invertase 1 (CINV1), nitrate reductase (NR) and H^+^-ATPase were subject to change by Fe deficiency in *Arabidopsis* roots. It is tempting to speculate that Fe deficiency induces the signaling cascades during phosphorylation processes at the plasma membrane level which are involved in interactions with 14-3-3 proteins^14^.

*Arabidopsis* genome contains thirteen expressed 14-3-3 genes and redundancy may exist between these isoforms^15,16^. According to amino acid sequence data and gene structure, the 14-3-3 members break into two major branches, the epsilon group (Epsilon, Iota, Mu, Omicron) and the non-epsilon group (Kappa, Lambda, Nu, Pi, Upsilon, Phi, Chi, Psi and Omega)^17^. The epsilon members are found in all organisms and are thought to be involved in basal eukaryotic 14-3-3 functions, while the non-epsilon group may be responsible for organism-specific regulatory aspects^15^. Both phosphate (P) and nitrate (N) deprivation cause isoform-specific and organ specific 14-3-3 transcript alterations. For instance, under phosphate efficiency, epsilon-group members are more affected compared to non-epsilon group members. Transcripts of *PSI*, *MU*, *OMICRON* and *PI* are decreased while *CHI*, *LAMBDA* and *KAPPA* are unchanged^18^. *KAPPA* expression is increased in leaves after K^+^-deprivation but not in roots and the expression of *CHI* did not change under nutrient deprivation^19^. Van Kleeff et al. conducted a series of growth experiments with higher order *Arabidopsis* 14-3-3 mutants and showed gene specificity and functional redundancy among non-epsilon group members in primary root elongation under control and abiotic stress conditions^20^. Previous studies have demonstrated that root growth, hormone sensitivity and more specifically the activation of a neutral cytosolic invertase, indeed showed both specificity and redundancy amongst 14-3-3 non-epsilon group members, *kappa*, *lambda*, *phi*, *chi*, *upsilon* and *nu*^20^*.* In addition, growing evidence demonstrates that 14-3-3 proteins have different affinities in the interaction with specific target proteins, what suggests that the large number of 14-3-3 isoforms in plants may reflect functional specificity. A large scale proteomics investigation showed that 14-3-3 target proteins bound differentially to 14-3-3 isoforms chi and epsilon during *Arabidopsis* seed development^21^. It has been proposed that the interaction specificity of certain 14-3-3 isoforms may involve the outer surface of 14-3-3, which shows variation between isoforms^22^. For instance, 14-3-3 isoforms from tobacco (*Nicotiana tabacum* L.) present a difference in affinity towards Sucrose-6-phosphate synthase (SPS) in the yeast two-hybrid system^23^. Compared to 14-3-3 isoforms belonging to the epsilon group, non-epsilon 14-3-3 isoforms were more active in the interaction and activation of H^+^-ATPase^24^.

The purpose of this paper is to address the question whether members of the non-epsilon group have functions in Fe-deficiency adaptation. Although the single mutant *grf11* (*omicron*) showed an Fe-deficiency phenotype^9^, a large number of 14-3-3 single mutant plants may lack a phenotype as other 14-3-3 member may take over the role of the mutated gene^20,25,26^. Therefore, we used higher order mutants which were obtained by combining the respective single mutants to overcome redundancy. Three higher order quadruple KOs, *kappa*/*lambda*/*phi*/*chi* (*klpc*), *kappa*/*lambda*/*upsilon*/*nu* (*klun*), and *upsilon*/*nu*/*phi*/*chi* (*unpc*) were generated and applied for physiological analysis. Here we investigated plant growth, Fe uptake, gene expression and 14-3-3 interactome to define the biological role of a sub-class of 14-3-3 (non-epsilon group) in the response of *A. thaliana* wild-type and 14-3-3 mutant plants to Fe deficiency.

## Results

### Plant growth and Fe concentration of Wt and the 14-3-3qKO lines under Fe deficiency

**Figure 1:**
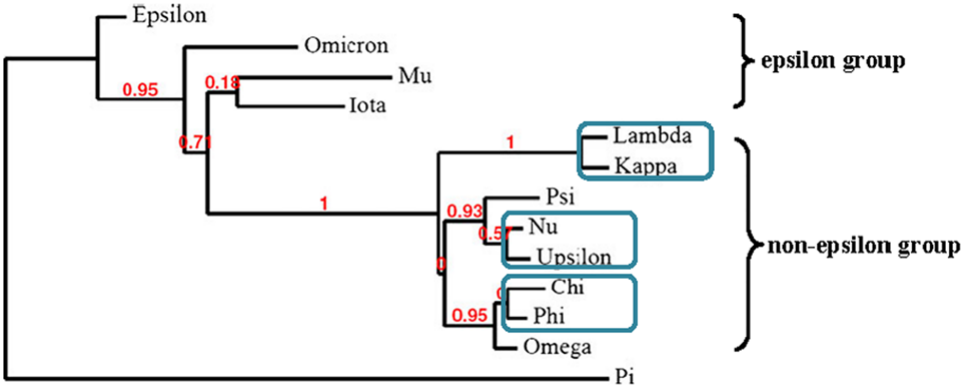
Phylogenetic tree of 14-3-3 superfamily in *Arabidopsis*. 14-3-3 s with the three closely related gene pairs of which T-DNA insertion lines are used in this study shown in blue boxes. The phylogenetic tree was constructed using the full-length amino acid sequence and Pi was used as outlier, the support branch values was marked in red. The division between the epsilon and non-epsilon group is indicated.

To investigate whether members of the non-epsilon group have a function in Fe-deficiency adaptation, three 14-3-3 quadruple KOs, *kappa*/*lambda*/*phi*/*chi* (*klpc*), *kappa*/*lambda*/*upsilon*/*nu* (*klun*), and *upsilon*/*nu*/*phi*/*chi* (*unpc*), were used in this study. The quadruple mutants were generated by crossing two double mutants: kl*pc, kl*un and un*pc. T-DNA insertions were monitored using PCR and the mutants were scored on full length 14-3-3 transcripts (for details see^20^). These six genes were chosen based on the phylogenetic tree that shows that these genes form three groups of closely related gene pairs as indicated in Fig. 1.

**Figure 2:**
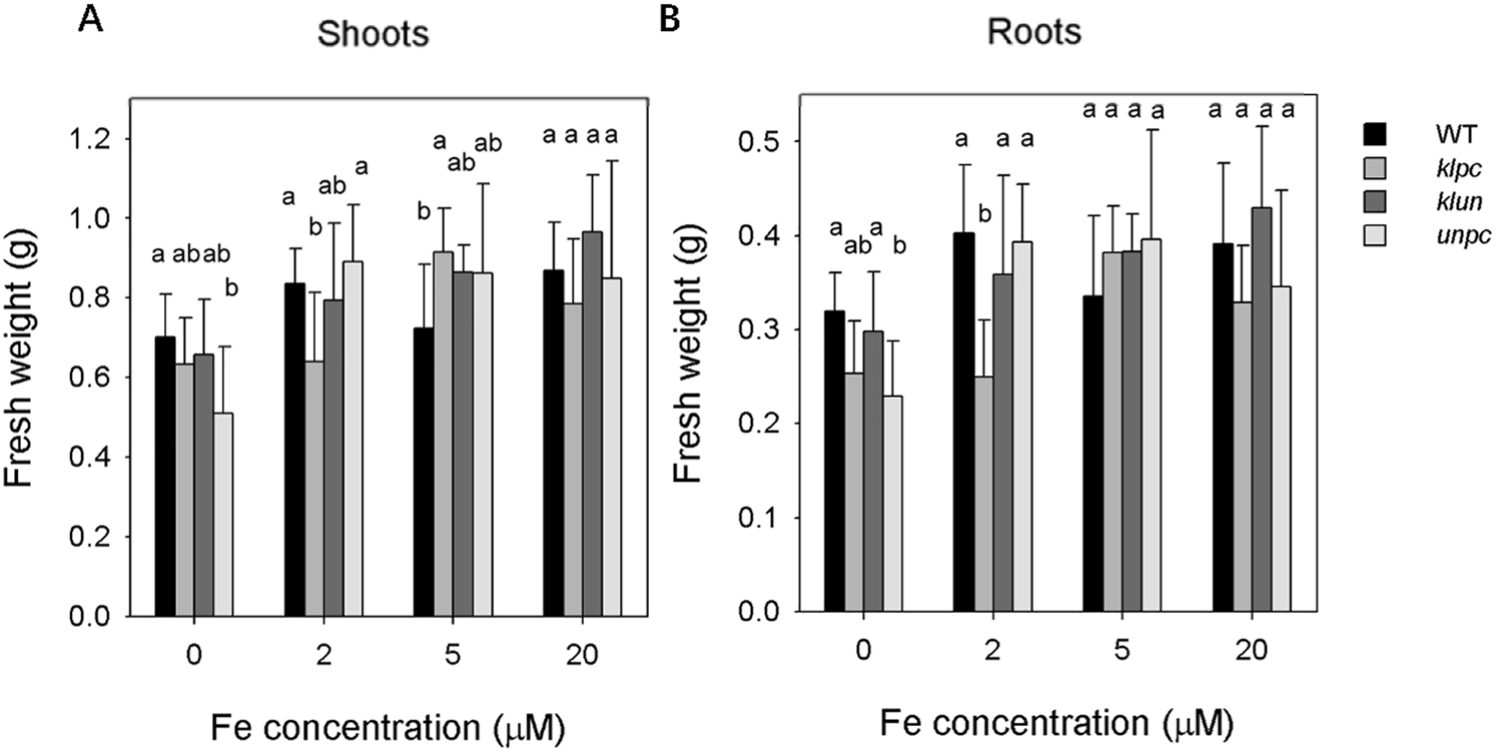
Growth performance of Wt and 14-3-3 qKOs under different Fe deficiency treatment. After two weeks of growth on ½ strength Hoagland solution, plants were grown for an additional 12 days at 0, 2, 5 and 20 μM Fe. Fresh weight of roots (**A**), and shoots (**B**) were measured from Wt and 14-3-3 qKOs plants. Values are means ± SD (n = 10). Different letters above the column indicate that the values are significantly different at p < 0.05 according to Tukey’s test.

To measure the effect of Fe deficiency on biomass production, wild-type (Wt) and 14-3-3 qKO plants were grown on hydroponics (½ strength Hoagland) with 20 µM Fe-EDTA for 2 weeks and then transferred to a medium with different Fe concentrations (0 µM, 2 µM, 5 µM and 20 µM) for 12 days before harvest. All 14-3-3 qKO plants maintained fresh weight production when grown at 5 µM Fe relative to plants grown under iron-sufficient condition (20 µM Fe), while the shoot weight of Wt was significantly lower compared to all the mutants (Fig. 2). At 0 µM Fe, all genotypes produced less biomass, whereat the fresh weight of both shoot and root of *unpc* were less than those of Wt. At 2 µM Fe, only *klpc* showed a significant growth reduction of both shoot and root (Fig. 2).

**Figure 3:**
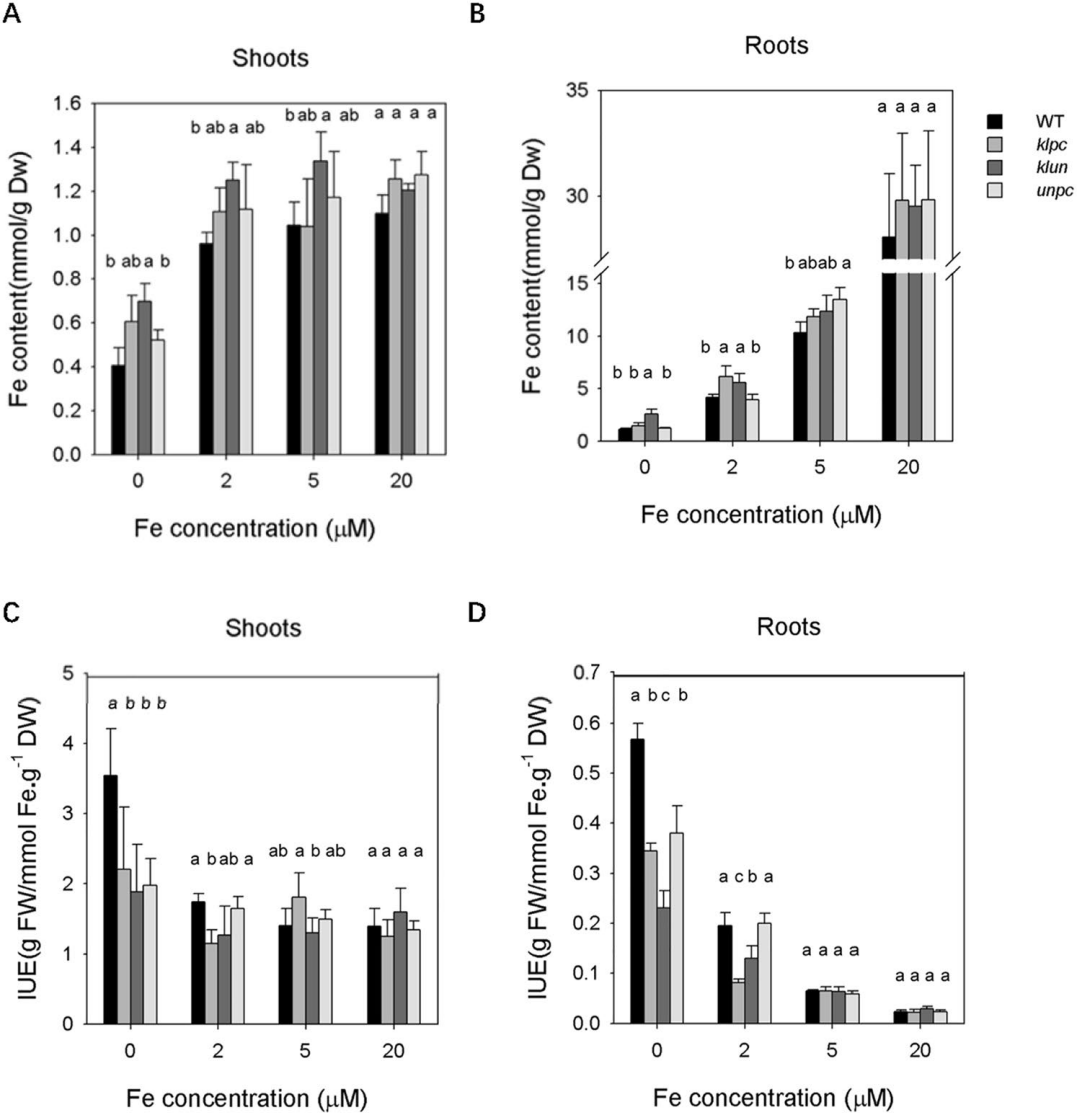
Fe content of shoots and roots of WT and 14-3-3 qKOs plants. After two weeks of growth on ½ strength Hoagland solution, plants were grown for an additional 12 days at 0, 2, 5 and 20 μM (= sufficient) Fe. Shoots and roots were harvested, weighed and pooled per two for the Fe-determination. (**A**) and (**B**), Fe-content of roots and shoots; (**C**) and (**D**), Iron Use Efficiency (IUE) of roots and shoots. Values are means ± SD (n = 5). Different letters above the column indicate that the values are significantly different at* p* < 0.05 according to Tukey’s test.

In the same plants as described above, the Fe contents of dry shoot and root material were measured. As expected, Fe shortage treatment reduced Fe content in the root and shoot tissues of all genotypes, with the greatest difference measured in the roots (Fig. 3). The comparison between genotypes showed that under iron-deficient condition (0 µM Fe-EDTA) and low-iron condition (2 µM Fe-EDTA), the Fe content in Wt shoots and roots were lower than those of *klun* while no difference was observed when plants were grown under iron-sufficient condition (20 μM Fe-EDTA). When plants were grown at ½ strength Hoagland with 0 µM and 2 µM Fe-EDTA, the Fe content in Wt shoots and roots did not differ from the Fe content in *unpc* (Fig. 3A,B). As a measure of Iron-Utilization Efficiency (IUE), we calculated the ratio between fresh weight and the Fe content of the shoot and root (Fig. 3C,D). Strikingly, the IUE of Wt shoot and root at 0 µM and 2 µM Fe-EDTA were much higher than those of *klpc* and *klun*, while *unpc* only showed significantly lower IUE than Wt when the plants were Fe starved (Fig. 3C,D).

### Root growth of Wt and 14-3-3 qKOs as affected by Fe-deficiency

**Figure 4:**
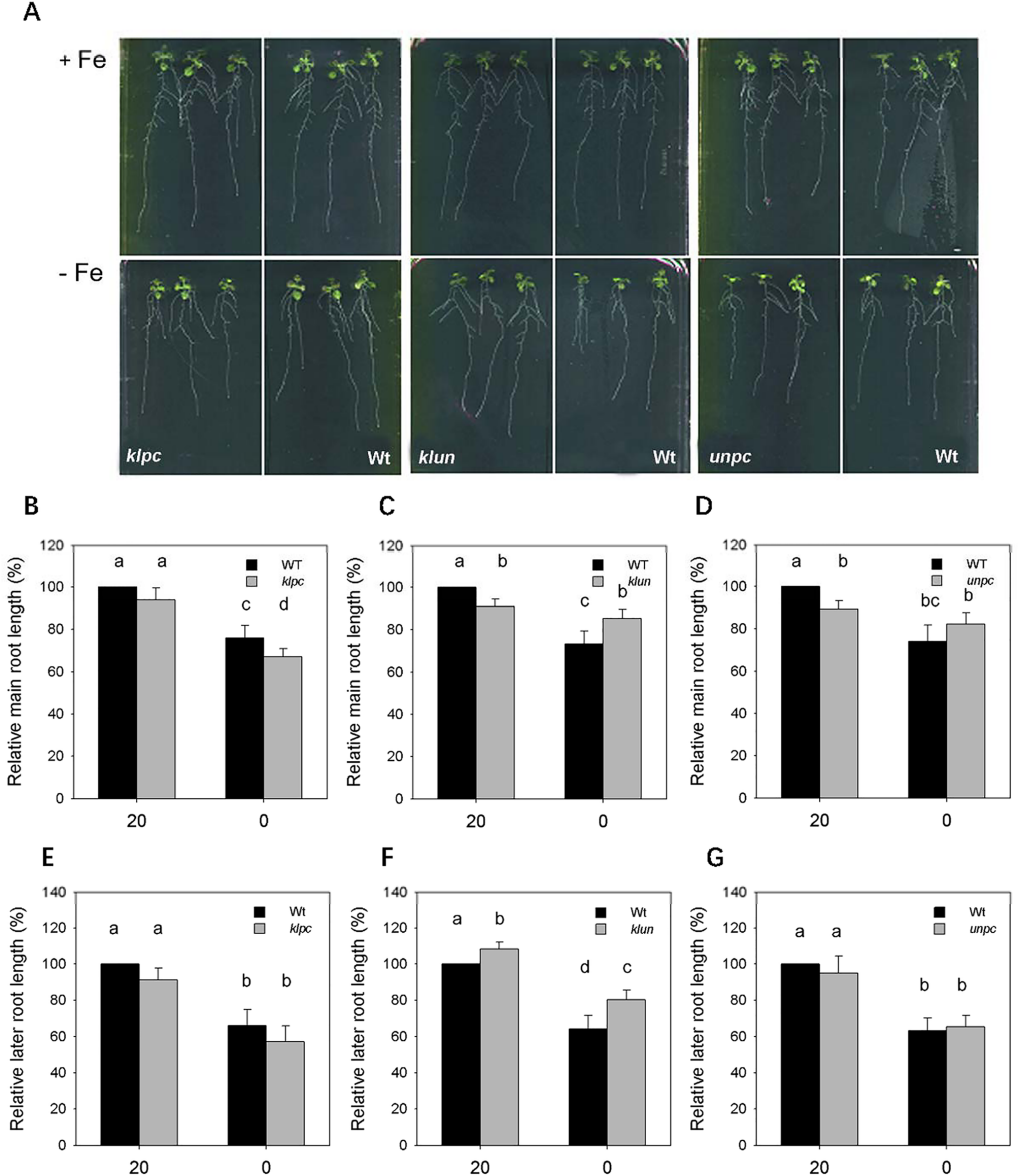
Primary root growth of Wt and 14-3-3 qKOs under Fe deficiency condition. (**A**) Wild-type and 14-3-3 qKOs seedlings were grown vertically on plates under long-day conditions and root growth was measured as described in the methods. (**B**-**D**) Length of the main root of Wt and 14-3-3 qKOs after 7 days grown under Fe deficiency condition. Primary root length of mutants was normalized to the value of the Wt under Fe-sufficient conditions. (**E**–**G**) Total lateral r root length of Wt and 14-3-3 qKOs after 7 days grown under Fe deficiency condition. Values are means of five independent experiments, and error bars represent ± SD (n = 5). Different letters above the column indicate that the values are significantly different at p < 0.05 according to Tukey’s test.

The reduction strategy can lead to an improved capacity for Fe uptake via inducing a series of root morphological changes, e.g. root hair length and root tip swelling^27^. Figure 4 shows root growth of mutant plants as compared with the Wt plants grown on the same agar plate. We observed that the growth of the main root of *klun* and *unpc* plants is less than that of Wt plants under Fe-sufficient condition (Fig. 4 B-D). Under Fe-deficient condition, the main root of *klpc* was shorter than that of Wt (Fig. 4B), while the main root length of *klun* was significantly longer as compared to Wt (Fig. 4C). There was no obvious difference in the main root length of *unpc* and Wt under Fe-deficient condition (Fig. 4D). We also measured the growth of lateral roots in the same 14-3-3 mutant plants. The total lateral root length of *klpc* and *unpc* were indistinguishable from that of WT plants, while total root length of lateral roots in *klun* plants was significantly longer than that of Wt plants under both Fe-sufficient and Fe-deficient condition (Fig. 4).

### Expression analysis of Fe deficiency-related genes in Wt and the 14-3-3qKO lines

**Figure 5:**
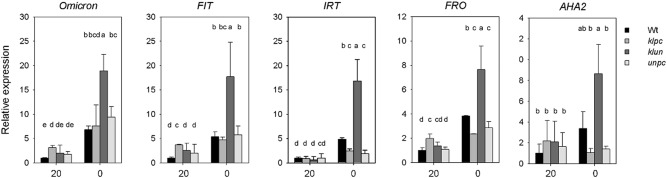
Expression analysis of 14-3-3 *Omicron*, *FIT*, *FRO*, *IRT* and *AHA2* in Wt and 14-3-3 qKO lines grown with (plus) and without (minus) Fe. *A. thaliana* seedlings were grown in 1/2 strength Hoagland with 20 μM Fe-EDTA for 22 days and then transferred to medium with or without 20 μM Fe-EDTA for 24 h. Expression levels were normalized to ubiquitin 10 (UBQ10) and then to the value of each gene from the Wt under Fe-sufficient conditions. Values are means of three independent experiments and error bars represent ± SD (n = 3). Different letters above the column indicate that the values are significantly different at *p* < 0.05 according to Tukey’s test.

*Arabidopsis* follows Strategy I for iron acquisition, involving rhizosphere acidification by plasma membrane H^+^-ATPases (AHA2), reduction of Fe (III) to Fe(II) performed by FRO2 and import of Fe(II) by the Fe transporter IRT1^28,29^. Whereas physiological changes are only manifest after long term Fe starvation, gene expression responds within 24 h^28,30^. Therefore, we measured the gene expression of *14-3-3 Omicron*, *FIT*, *FRO2*, *IRT1* and *AHA2* in roots of Wt and 14-3-3 qKOs after 24 h Fe deficiency by quantitative real-time PCR (qPCR) (Fig. 5). In Wt plants, the 24 h Fe starvation induced increases in the transcription levels of *Omicron*, *FIT*, *GRF11*, and *FRO*, but not *AHA2*. Besides, the transcriptional level of *Omicron*, *FIT*, and *FRO* in *klpc* mutant was higher compared to Wt under Fe sufficient condition, while the expression level of *IRT* and *FRO* was lower than that of Wt. The most obvious effect of Fe deficiency on gene expression was shown by the *klun* plants: *klun* mutants showed significantly higher expression of *Omicron*, *FIT*, *GRF11*, and *FRO* as compared to Wt when the plants were Fe starved (0 μM), but these differences disappeared under Fe sufficient conditions (20 μM) (Fig. 5). This suggests that somehow the 14-3-3*Kappa/Lambda/Upsilon/Nu* proteins act as negative regulators of gene expression when the plants are Fe-starved.

### Quantitative affinity-purification mass spectrometry analysis of the 14-3-3 interactome of Wt roots as affected by Fe deficiency

In view of the reported Fe deficiency-induced changes in protein amounts^11^ and phosphorylation^13^, we addressed the question whether and how Fe-deficiency affects the root 14-3-3 interactome, which is phosphorylation dependent. To this end, we carried out a semi-quantitative interactome analysis by affinity-purification mass spectrometry (qAP-MS) to assess Fe deficiency induced changes in 14-3-3/target interaction. To exclude proteins that bind non-specifically to the beads, we also performed a mock pull-down with empty beads along with the test pull-down experiments (Fig. S1). In this experiment, we included the “unused” values generated from the software ProteinPilot (version 3.0; Applied Biosystems, Foster City, CA, USA; MDS Sciex, https://sciex.com/products/software/proteinpilot-software)^31^. After removal of contaminant and false positive interactors (for details see M&M), a curated list of 117 proteins was identified in the pull-down with either Wt or *klun* roots protein extract, with unused value > 2 in at least 2 independent samples remained (Table S1). These proteins are either so-called primary interactors (bind directly to 14-3-3) or secondary interactors (part of a multiprotein complex of which one protein is a primary interactor). A comparison between our results (Table S1) and published 14-3-3 interactome studies shows that 24 proteins identified in this study have been previously reported as 14-3-3 interactors interactors^19,21,32,33^ , such as Glucose-6-phosphate 1-dehydrogenase 3 (G6PD3), ATP synthase (ATP1, ATP5). 16 proteins from our list were reported as Fe-responsive proteins, amongst which several enzymes involved in S-adenosylmethionine synthesis, two cytosolic invertases, Ferretin-1, germin-like protein GLP5 (a plasmodesmata-localized protein involved in the regulation of primary root growth)^34^ and others^11,13,35^. We assigned the identified binding proteins to different KEGG categories, as shown in Fig. S2. The majority of the identified proteins are related to “carbohydrate and energy metabolism”, “amino acid metabolism”, “protein folding, sorting and degradation”, and “transport and catabolism” (Table S2). As will be discussed later, the 14-3-3 interactome is not a random collection of proteins, but consists of distinct networks of interrelated proteins and protein complexes, involved in glycolysis, methionine metabolism, oxidative phosphorylation. Further, three major protein complexes were identified based on known and predicted interactions: the head structure of the FoF1-synthase and vacuolar V-ATPase, tubulins and the TCP-1/cpn60/HSP chaperones, as well as four functional networks: elongation factors, chaperones, glycolysis and TCA cycle and cysteine/methionine metabolism (Table S3).

### Comparison of 14-3-3 interactome under control and Fe deficient conditions

**Figure 6:**
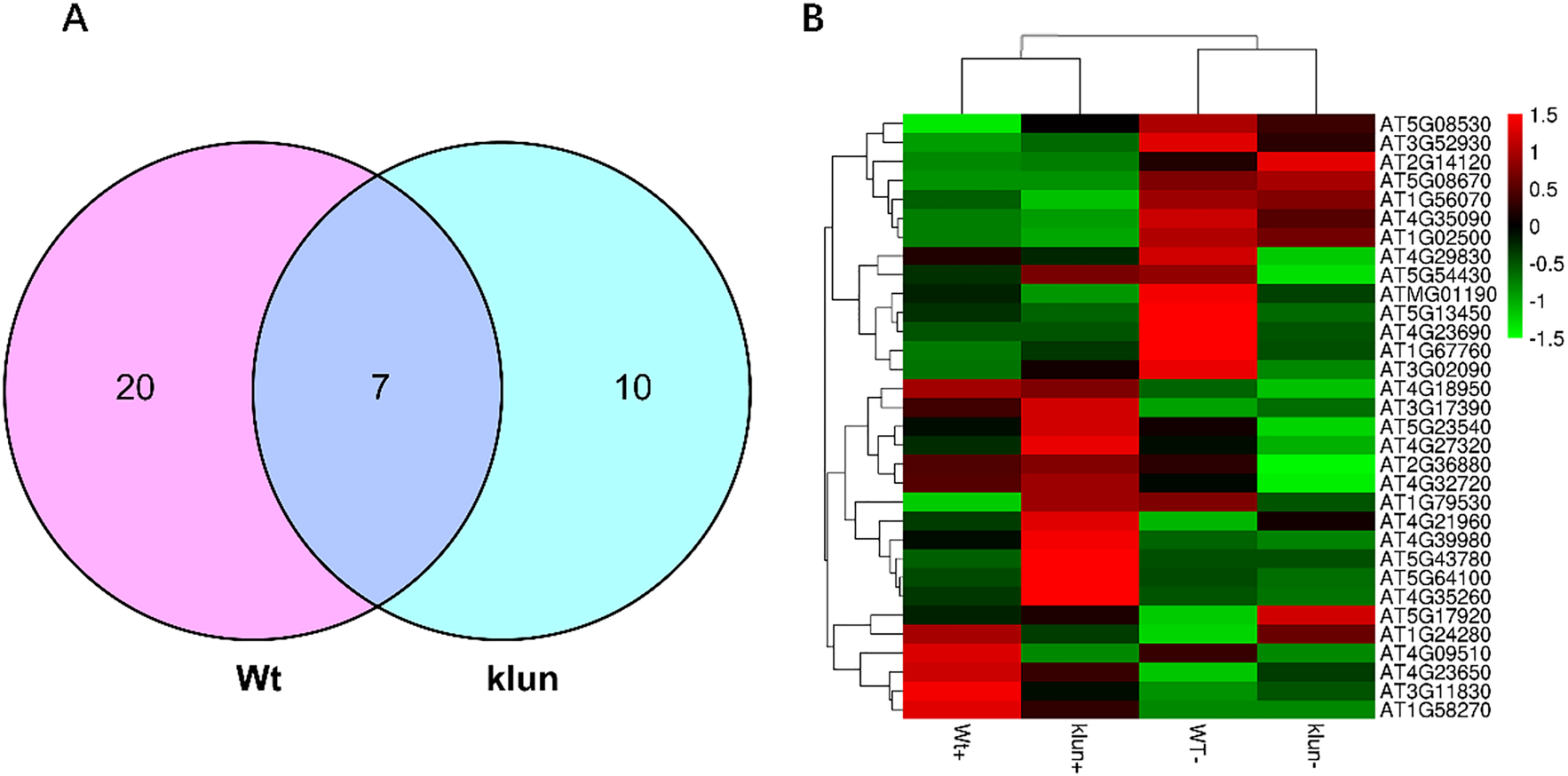
The changes of 14-3-3 interaction in Wt roots or *klun* roots induced by Fe-deficiency. (**A**) The Venn diagram of changed interactors identified in the comparison between Fe-sufficient and Fe-deficient condition in Wt and klun. **B** Heatmap of changed interactors identified in the comparison between Fe-sufficient and Fe-deficient condition in Wt and klun pull-down experiment.

To study the changes of 14-3-3-target interaction in response to Fe deficiency, we further analysed the abundance of binding proteins identified from the pull-down experiment conducted with roots extract under Fe-sufficient or Fe-deficient condition in both Wt and *klun* plants, based on normalized intensity-based absolute quantification (iBAQ) values (normalized to the bait proteins in that run). The iBAQ intensities act as a measure of protein abundance and can be used to compare protein abundance between different samples. Among the 117 identified proteins, 27 and 17 proteins were significantly changed upon Fe deficiency in Wt and *klun*, respectively (Tables S4 & S5). According to the Venn analysis, 21 and 10 proteins were uniquely changed in Wt and *klun* pull-down experiment, respectively (Fig. 6A). Moreover, we found 7 proteins that were commonly changed upon Fe deficiency in both the Wt and *klun* pull-down experiment, and most of them showed similar trends in *klun* as well as those in Wt upon Fe deficiency (Tables S4 & S5). To visualize the entire data set, a heatmap was generated to represent the normalized abundance of proteins in each pull-down experiment (Fig. 6B). A total of 37 14-3-3 binding proteins showed clear separation between pull-down experiment on plants grown under Fe-deficient and Fe-sufficient condition (Table S6). Especially, S-adenosylmethionine synthase 1 (MAT1, AT1G02500), Elongation factor EF-2 (LOS1, AT1G56070), ATP synthase subunit alpha (ATP1, ATMG01190), and mitochondrial ion transporting ATP synthase beta-subunit (AT5G08670) were significant enhanced in their interaction with 14-3-3 proteins in both Wt and *klun* Fe-deficient plants (Table S6). Five proteins, including mitochondrial-processing peptidase subunit beta (MPPBETA, AT3G02090), ATP synthase subunit delta (ATP5, AT5G13450), TCP-1/cpn60 chaperonin family protein (AT1G67760), PHOS32 (AT5G54430) and Dirigent protein 6 (AT4G23690) solely increased in the pull-down with extract of Wt plants upon Fe deficiency. Moreover, the abundance of Glucose-6-phosphate 1-dehydrogenase 3 (G6PD3, AT1G24280), Neutral invertase 2 (CINV2, AT4G09510), Calcium-dependent protein kinase 3 (CDPK6, AT4G23650) and ZW9 (AT1G58270) were uniquely reduced in the pull-down from extracts of Wt plants grown under Fe-deficient conditions (Table S6). KEGG pathway enrichment analysis was also conducted with the changed binding proteins from the aforementioned comparisons (Fig. 6). The enriched KEGG pathways with the highest representation of the changed proteins in both Wt and *klun* were mostly associated with amino acids metabolism and carbohydrate and energy metabolism, such as “Cysteine and methionine metabolism” (ko00270), “Oxidative phosphorylation” (ko00190), and “Glycolysis / Gluconeogenesis” (ko00010) (Table S7 & S8).

**Figure 7:**
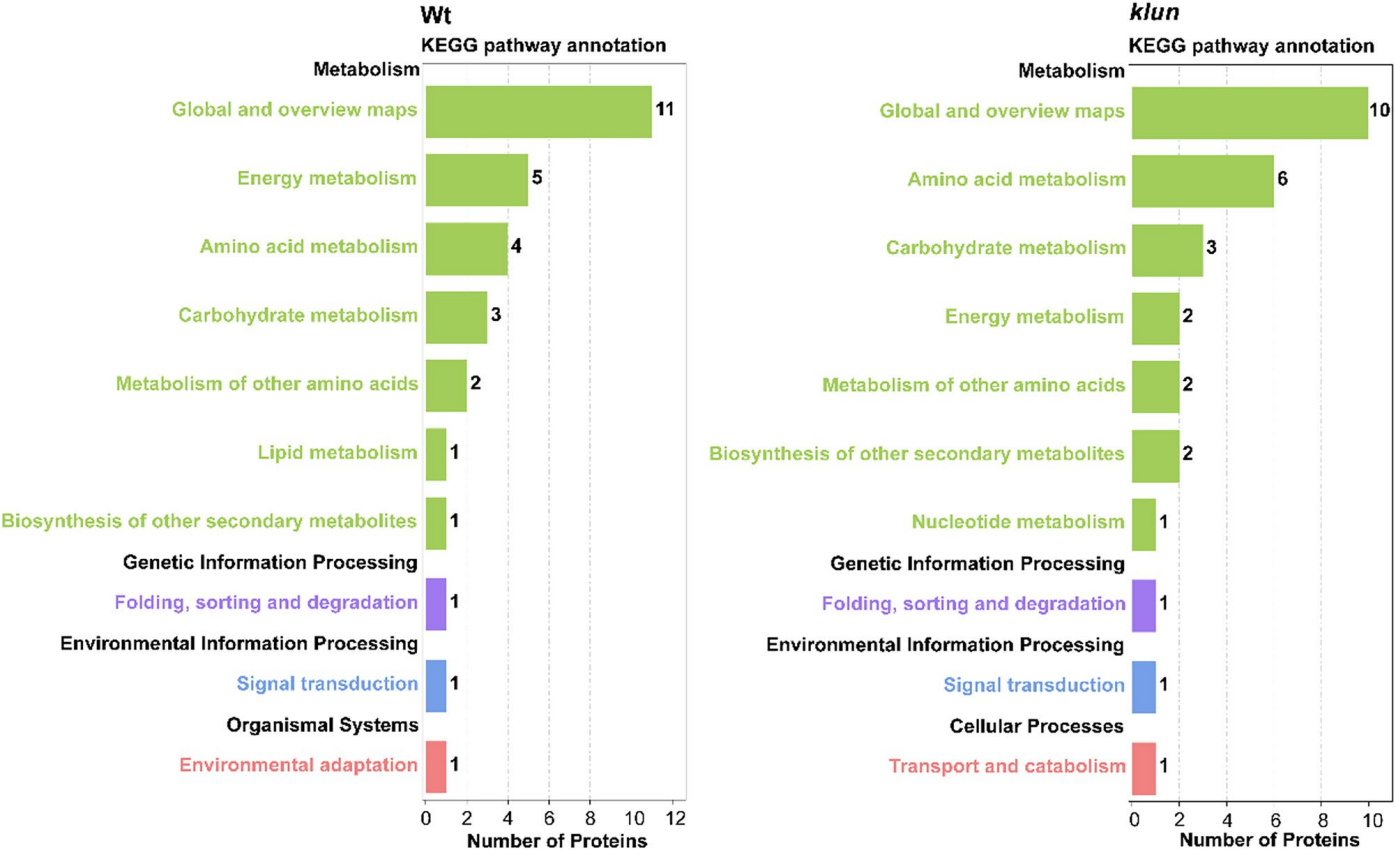
KEGG pathway enrichment analysis of 14-3-3 potential target proteins changed upon Fe deficiency in Wt and *klun* roots. The left y-axis indicates the KEGG's A-level and B-level categories. The black font is the A-level category name, and the color font is the B-level category name; x-axis indicates the number of proteins in the corresponding B-level classification. See also Table S7 & S8 showing the KEGG enrichment analysis of the identified proteins. KEGG pathway annotation was obtained from the KEGG pathway database (http://www.genome.jp/kegg/pathway).

Comparison of the *klun* interactome with that of Wt plants grown under Fe-sufficient condition, showed significant quantitative differences in the pull-down from the two genotypes (Fig. 7). The *klun* interactome lacks 7 proteins of the Wt interactome and vice versa the Wt interactome lacks 4 proteins of the *klun* interactome. Moreover, 17 proteins are 3- to fourfold enriched in the *klun* interactome as compared to Wt, whereas only 3 proteins are enriched in the Wt interactome as compared to *klun* (Table S6). So, the absence of the four 14-3-3 isoforms in the *klun* mutant has a clear impact on the 14-3-3 interactome and it is noteworthy that identified proteins are enriched in the *klun* interactome, what cannot be the result of a loss of in vivo phosphorylation in the absence of the four 14-3-3 proteins.

A. The Venn diagram of changed interactors identified in the comparison between Fe-sufficient and Fe-deficient condition in Wt and *klun*. B. Heatmap of changed interactors identified in the comparison between Fe-sufficient and Fe-deficient condition in Wt and *klun* pull-down experiment.

## Discussion

14-3-3 Proteins regulate the activities of a wide array of targets via direct protein-protein interactions and play a crucial role in many metabolic pathways^36^. Several studies have implied that 14-3-3 proteins have crucial roles in Fe deficiency However, analysis of the function of 14-3-3 proteins through mutant phenotyping is a challenging task due to gene specificity and functional redundancy as previously reported^18,20,24^. To avoid missing the phenotype of mutants due to redundancy we studied three high order mutants (*klpc*, *klun* and *unpc*) from the non-epsilon group which were generated by crossing two double mutants: kl*pc, kl*un and un*pc. In this study, we found the distinct physiological responses to Fe deficiency of Wt and the three 14-3-3 quadruple mutant lines and conducted a qAP-MS to analyze the 14-3-3 interactomes of roots in response in both Wt and *klun*.

Fe deficiency is a major nutritional disorder that causes decreases in vegetative growth and marked yield and quality losses^37^. In this study, the 14-3-3 qKOs performed distinctly in response to long-term Fe deficiency at physiological levels. Under Fe-deficient condition, *unpc* showed the strongest growth reduction of shoot and root, and growth of *klpc* showed a significant reduction of both shoot and root under low Fe condition (Fig. 2). The uptake of Fe by *klun* is clearly better than that of Wt (Fig. 3A,B). Nevertheless, Fe deficiency induced a much higher IUE (Iron Use Efficiency) of Wt shoot and root than that of the 14-3-3 mutants (Fig. 3C,D), what points to a more efficient use of available Fe in the Wt plants and thus to a role for 14-3-3 proteins in Fe-translocation and/or use after uptake from growth medium.

Studies on the micronutrient requirements of the rhizosphere suggest that root morphology (e.g. root length, root hair density) are critical for plants to acquire available Fe^38^. Iron-deficient plants must allocate their limited energy resources between finding available iron and physiological maintenance. Low Fe bioavailability induce plants to address their efforts to acquire the nutrient by increasing the root surface. Our results indicated that the length of both main and lateral roots in *klun* was significantly longer than those of Wt plants under Fe-deficient condition (Fig. 4). The increased root length of *klun* were likely correlated to the higher Fe uptake. In addition, we found that the main root length of *klun* and *unpc* was shorter under Fe-sufficient condition as compared to Wt. According to our data, the mutant plants that combine *kl* with *un* (*klun*) showed a better Fe uptake and longer root length as compared with Wt at Fe deficiency, while these phenotypes were absent in mutant plants that combine *un* with *pc* (*unpc*). This indicates that the combination of kappa and lambda present isoform specificity amongst the 14-3-3 genes tested. Whether there is redundancy between kappa and lambda requires further testing by analyzing the single and double mutants.

Along with the diverse physiological responses to Fe deficiency observed in 14-3-3 qKOs, changes in gene expression related to physiological responses were also observed in the present study. H^+^-ATPases (AHA2), FIT, FRO2 and IRT1 together mediate Fe deficiency induced medium acidification and Fe uptake in Strategy I plants^39–41^. In this study, the expression of *14-3-3 OMICRON*, *FIT*, *FRO2* and *IRT1* were all induced by Fe deficiency, whereat the induction in *klun* mutant was strongest among all the tested genotypes (Fig. 5). Under Fe deficiency, high-level expression of the genes encoding the abovementioned proteins is controlled by the bHLH transcription factor FIT, where the induction of FIT expression is dependent on the 14-3-3 Omicron protein^9^^.^ In a detailed microarray analysis, GRF11 was significantly induced in the root elongation and maturation zone after subjecting plants to Fe deficiency for 24 h^42^. Loss of function of GRF11(14-3-3 Omicron) resulted in failure of acidification and ferric chelate reductase (FCR) induction, and thus decreased Fe uptake. However, another two 14-3-3 isoforms, GRF9 (14-3-3 Mu, Epsilon group member) and GRF1 (14-3-3 Chi, non-Epsilon group member) did not show a noticeable difference in expression in a Northern analyses in *Arabidopsis* upon iron deficiency^18^. Although we observed that the 14-3-3 qKOs affected the expression of Fe deficiency responding genes, it remains a question whether this regulation is affected directly by the mutations or indirectly by the 14-3-3 OMICRON taking over other’s function (redundancy). Moreover, combining *un* with *pc* (*unpc*) and *kl* with *pc* (*klpc*) showed no different effect on expression level of 1*4-3-3 omicron*, *FIT*, and *AHA2* as compared with WT under Fe deficiency, raising the possibility that KL but not UN isoforms are involved in the iron deficient phenotype (Fig. 5).

Our results from the pull-down experiments using recombinant 14-3-3 s and protein extract from Fe-deficient or Fe-sufficient roots showed a total of 117 proteins identified as putative 14-3-3 clients. It must be noted that proteins found in the 14-3-3 s affinity purification include not only primary or direct 14-3-3 targets but also secondary or indirect targets as members of multi protein complexes containing 14-3-3 s. Of these, 24 proteins have been identified in other 14-3-3 s interactome studies or characterized as 14-3-3 binding targets in vivo or in vitro, such as EIF4A (AT3G13920)^43^, CINV1 (AT1G35580)^44^ and CDPK6^45^. In addition, 16 proteins were reported as Fe-responsive proteins including a fructose-bisphosphate aldolase (AT3G52930), two cytosolic invertases (AT1G35580, AT4G34860), a 5-methyltetrahydropteroyltriglutamate-homocysteine methyltransferase (AT5G17920), and two S-adenosylmethionine synthase (AT1G02500, AT2G36880)^11,35,46^ (Table S1). According to KEGG classification, the most significantly enriched terms were associated with “amino acid metabolism”, “carbohydrate and energy metabolism”, “protein folding, sorting and degradation”, and “transport and catabolism” (Fig. S2), which are consistent with previous studies on 14-3-3 interactomics^21,32,47^.

Since most proteins were found with high abundance in all pull-down experiments, it is difficult to directly assess the dynamic response of 14-3-3 interactions to Fe deficiency. Thus, quantitative analysis of the identified 14-3-3 clients is essential to reveal responses to Fe deficiency. By comparing the normalized iBAQ, we found that 27 and 17 proteins were significantly changed in the 14-3-3 interactome of Wt and *klun* upon Fe deficiency treatment, respectively (Tables S4 & S5). Moreover, a comparison of the Wt and *klun* interactome of Fe sufficient roots showed a quantitative or absolute difference in the interaction of 32 proteins (Table S6). So, the 14-3-3 interactome was clearly affected by Fe deficiency (Wt, ± Fe) as well as by the absence of four 14-3-3 isoforms (Wt/*klun*, + Fe). The observed changes are mainly in proteins with a function in carbohydrate and energy metabolism and cysteine/methionine synthesis and we will therefore limit our discussion to these pathways (Fig. S2). In this study, changes of protein abundance in carbohydrate and energy metabolism enzymes upon Fe deficiency are consistently identified among different proteomics studies^11,46^. For example, G6PD3 (AT1G24280)(down-regulated, 0.31-fold) and fructose-bisphosphate aldolase (AT3G52930) (up-regulated, 4.03-fold) was significantly changed in the pull-down with Wt root upon Fe deficiency, whose abundance have shown the similar trends (0.60-fold and 1.41-fold, respectively) in iTRAQ protein profile analysis of Fe-deficient *Arabidopsis* roots^11^ (Tables S5 & S6). An increase in NADH^+^ ATP production by activation of the glycolytic pathway and mitochondrial respiration can provide the reducing equivalents to keep the Fe (III) reductase working and fuel the plasma membrane ATPase: processes that are essential for the Fe-uptake mechanism in iron-deficient roots^48^. In this study, we found that three subunits of the mitochondrial ATP synthase head structure (α, β and δ), NADH dehydrogenase and the mitochondrial-processing peptidase MPPBETA. FoF1-synthase has been reported as 14-3-3 target^49,50^ and the identification of only subunits of the head-structure is in line with the evidence that the β-subunit is the direct target for 14-3-3. Functionally, the mitochondrial synthase activity is reduced by interaction with 14-3-3^49^ and in that respect it is surprising that in Wt, Fe deficiency enhances the interaction with the FoF1-subunits and the mitochondrial processing peptidase 2- to tenfold (Table S5). This increased interaction suggests a down-regulation of the ATP-synthase, what is contrary to the expected increase in respiration. Interaction with MPPBETA and CI51 is increased 3- to tenfold respectively and this warrant further study of the functional consequences of 14-3-3/MPPBETA and CI51 interaction. It should be noted that the related head structure of the vacuolar V-ATPase (A, B and E1 subunits) was isolated as well, but Fe deficiency had no effect on the interaction with the V-ATPase.

Methionine synthesis is important in the Fe deficiency response as it provides the precursors for ethylene and nicotianamine (NA), an important chelator with a crucial function role in Fe homeostasis and transport^51,52^. Fe deficiency induced multiple S-adenosylmethionine synthases (gene expression and protein amount) involved in S-adenosyl-Met^53^ biosynthesis, an important precursor for ethylene production^11^. 14-3-3 s are linked to the ethylene biosynthesis by interacting with S-adenosylmethionine (SAM) synthase^53^, ACC synthase and 1-aminocyclopropane-1-carboxylate synthase (ACS)^19,32,54,55^. Here, we identified four S-adenosylmethionine synthases (MAT1, MAT2, MAT3, and MAT4) and the 5-methyltetrahydropteroyltriglutamate-homocysteine methyltransferase (ATMS1) in the 14-3-3 s interactome, underlining the importance of 14-3-3 s in this pathway (Table S1 and S2). In addition, SAM synthases MAT1, MAT2 and MAT3 were found to be significantly increased at the protein level in the presence of iron deficiency in *Arabidopsis* roots^11,56^, while our data suggested that Fe-deficiency stimulated the interaction of MAT1/14-3-3 s, but reduced the interaction with MAT4 in Wt and *klun* (Tables S5 & S6). It may imply that the interaction between 14-3-3 and adenosylmethionine synthetases involved in multiple molecular mechanisms underlying the Fe deficient stress responses, and it is of prime importance to address the question how 14-3-3 s affect the SAM-synthase activities. Amongst the proteins that were lost in the 14-3-3 pull-down with protein extract form Wt plants, ECIP1 (EIN2 C-terminus interacting protein 1) may be relevant in view of the role of ethylene in Fe deficiency adaptation. ECIP1 is an MA3 domain-containing protein that interacts with EIN2, a central membrane protein that acts downstream of ethylene receptors, and upstream of ethylene regulated transcription factors. ECIP1 directly interacts with EIN2 and loss-of-function of ECIP1 resulted in enhanced ethylene response^57^. If ECIP1/14-3-3 interaction prevents ECIP1 breakdown as is e.g. the case for ABF3 , then loss of interaction between ECIP1 and 14-3-3 will result in enhanced ethylene signaling. So, this warrants further investigation.

Fe deficiency may cause changes in post-translational modifications as well. Several protein kinases (e.g. MPK3/MPK6) accumulate differentially upon Fe-deficient plants, what suggests that alterations in protein phosphorylation induced by Fe-deficiency are involved in Fe homeostasis^58^. In this study, we found that the interaction of 14-3-3/CDPK6 (AT4G23650) only significantly decreased accumulation in Wt upon Fe deficiency, suggesting that phosphorylation level of 14-3-3 binding targets is different under Fe-deficient conditions, as reported for CINV1, AHA1 and MAPKKK^13^. As the changes in 14-3-3 interaction may be due to changes in protein abundance and protein phosphorylation, it is difficult to predict how the absence of four 14-3-3 proteins, as is the case in the *klun* mutant, will affect the interactome. It may reduce in vitro interaction because in vivo binding of 14-3-3 proteins to a phosphorylated motif in target proteins enhances the in vivo level of phosphorylation as the site is then protected from dephosphorylation by phosphatases. On the other hand, the absence of 14-3-3 s may result in more target protein in the pull-down because 14-3-3 can act as repressors of transcription factors or kinases. An example of the latter is the suppression of SOS2 activity by 14-3-3 binding^59^. Thus, quantification of abundance as well as phosphorylation level of the 117 14-3-3 putative binding proteins in the respective input fractions needs to be done in the future.

Altogether, these findings provide novel insights into the role of 14-3-3 non-epsilon group in response of Fe deficiency. Our data highlight that the combination of kappa and lambda presents isoform specificity in iron deficient phenotype. In addition, the absence of the four 14-3-3 isoforms in the *klun* mutant has a clear impact on the 14-3-3 interactome upon Fe deficiency. This work has greatly reduced the scope of research objects for follow-up work towards the role of tested 14-3-3 isoforms in Fe acquisition. Analysis of single, double mutants of *k* and *l* will reveal whether redundancy exists for the observed phenotypes. In addition, we found that the interaction of 14-3-3/ SAM synthases plays an essential role in plants responding to Fe deficiency. How 14-3-3 s affect the SAM-synthase activities and thereby regulate the ethylene signaling pathway needs to be addressed.

## Materials and methods

### Plant growth conditions

All plants used are in the *Arabidopsis thaliana* Columbia ecotype (Col-0) background. The 14-3-3 quadruple KO mutants were generated by crossing the double mutants as described in the previous study reported by van Kleeff et al.^20^. The mutant seeds collection and plants growth were carried out with the permission from the Vrije University Amsterdam. Experimental research using plants comply with institutional, national, or international guidelines. Plants were grown in ½ strength Hoagland solution (pH 5.8) with 20 μM Fe(III)-EDTA in a growth chamber at 14/10 h day/night regime, 22/18 °C day/night temperature and a photon flux density of 170 μmol•m-2•s-1. After 22 days of germination, plant roots were washed with once with 10 mM EDTA for 10 min followed by two times wash in Milli Q, before transfer to either iron-sufficient (20 μM Fe(III)-EDTA) or iron-deficient (iron omitted) culture medium for 24 h. Then the roots were harvested and immediately cleaned with Milli Q. After that, the roots were dried on tissue paper and snap frozen in liquid nitrogen. Total roots were ground in liquid nitrogen and weighed, and stored at -80 °C. The plant material was used for 14-3-3 pull-down experiments. For growth and Fe content measurement, plants of Wt and 14-3-3 qKO plants were grown in ½ strength Hoagland solution (pH 5.8) with 20 μM Fe(III)-EDTA for 14 days, then young seedlings were transplanted to a new medium supplemented with 0, 2, 5, and 20 μM Fe(III)-EDTA. Leaves and roots were harvested 12 days after Fe deficient treatment. To get rid of surface constituents, the roots were washed in 10 mM EDTA for 10 min and then rinsed twice in Milli-Q before harvest. The collected shoots and roots from two plants were pooled for each biological replicate and harvested separately and dried at 150 °C for 2 days.

### Analysis of the Fe content in Arabidopsis shoots and roots

Fe content in Arabidopsis leaves and roots was determined by the BPDS (bathophenanthrolinedisulfonicacid) method as described previously by Schmidt^60^. In brief, 4 to 8 mg dried sample was well mixed in 2 mL Eppendorf tubes and heated at 95 °C in 75 µL nitric acid (65%) for 6 h. After the samples were completely digested, 50 µL of H_2_O_2_ (30%) was added, and the solution was incubated at 56 °C for 2 h. The volume was adjusted to 200 µL with sterile water. 20 µL of this solution was diluted in 980 µL of BPDS buffer (1 mM BPDS, 0.6 M sodium acetate, and 0.48 M hydroxylammonium chloride). The concentration of Fe-BPDS was measured at 535 nm. A standard curve was prepared by dilution of a stock FeSO_4_ solution dissolved in 0.1 M HCl. The iron utilization efficiency (IUE) was calculated based on Fe and dry weight accumulation, as described by Fageria and Baligar^61^.

### Root phenotyping

Seed sterilization and germination was according to our previous study^20^. For the root phenotyping, seeds were germinated on 120 mm × 120 mm petri dishes containing 0.5 × MS medium (pH 5.8) solidified with 12 g/L of plant agar (Sigma A1296) after three days of stratification. After 4 days, three Wt and three mutant plants were transferred to new plates containing Fe-deficient or Fe-sufficient medium and grown vertically. Plates were scanned using a flatbed scanner after 7 days and the images were analyzed using EZ-Rhizo^62^. The parameters chosen in this study are: 1) main root length, 2) Total length of the lateral roots.

### Real-time PCR assay

Total RNA was extracted from root tissues using the NucleoSpin® RNA Plant Kit (MACHEREY-NAGEL), and first-strand cDNA was synthesized from 2 μg of total RNA using the Superscript II Kit (Invitrogen, USA) with oligo d(T)18 primers according to the manufacturer’s instructions. Quantitative RT-PCR reaction contained 100 ng cDNA, 1 pmol of each primer, 2 × Sybr Green PCR buffer (Bio-Rad, Hercules). The PCR conditions for the amplification of *14-3-3 omicron*, *FIT*, *FRO2*, *IRT1*, *AHA2* and *ubiquitin 10* were as follows: 1 min at 94 °C, followed by 30 cycles of 45 s at 94 °C, 60 s at 54 °C and 75 s at 72 °C. The PCR products were examined according the 2-∆∆CT method. Each sample was assayed three times. The relative expression was calculated against that of the internal control gene ubiquitin 10 (UBQ10). Primer pairs used for each gene are listed in Table S9.

### Affinity purification of 14-3-3 target proteins

Recombinant His-tagged proteins were purified as described in^44^. Protein concentrations were determined by Bradford micro-assay (Bio-Rad) using BSA as a standard. Arabidopsis roots from WT and *klun* plants were ground in liquid nitrogen and extracted with extraction buffer (50 mM HEPES-NaOH (pH 7), 10 mM MgCl_2_, 1 mM Na_2_EDTA, 2 mM DTT, 10% ethylene glycol, 0.02% Triton, 1 × protease inhibitor cocktail and 1 × phosSTOP). Protein extracts were centrifuged twice at 20,000 g for 15 min. To avoid the isoform specifically binding of the 14-3-3 target proteins, equal amount of four 14-3-3 isoforms (His-KAPPA, His-LAMBDA, His-NU, His-UPSILON) were well mixed and then coated to PureProteome Nickel magnetic Beads (Millipore) according to manufactures’ protocol. Protein extracts were collected from roots of *klun* and WT treated with 0 and 20 μM Fe (III)-EDTA for 24 h. After coating of 40 µg of His-14-3-3 to 100 µl nickel beads and extensive washing with binding buffer (50 mM sodium phosphate, 300 mM sodium chloride, 10 mM imidazole, pH 8), 2 mg protein extract was added to the 14-3-3 beads and incubated overnight at 4 ºC. To distinguish background proteins, we performed mock pull-down with empty beads, where the extracts were incubated with beads that were not coated with His-14-3-3. Beads were extensively washed in 1 ml wash buffer containing 10 mM imidazole for 5 min and this was repeated 5 times. The bound protein complexes were eluted off from beads with 100 μl of wash buffer containing 100 mM imidazole for 20 min. The experiment was conducted three times with independent biological replicates, meaning that in the end each genotype had 6 profiles. CBB-stained gel was used as the loading control (Fig. S1). The affinity-enriched proteins were separated on SDS-PAGE, and then characterized by LC-MS-MS semi-quantitatively.

### In-gel digestion and LC-MS/MS analysis

LC-MS/MS Analysis and Comparative Proteomic Analysis Proteins from pull-down experiments were resolved on a one-dimensional 10% SDS polyacrylamide gel. After staining with Coomassie Blue, each sample lane was divided into four pieces. Each gel slice was cut into small particles and transferred to a clean micro-centrifuge tube. For in-gel digestion, 50% acetonitrile containing 50 mM (NH_4_)HCO_3_ was added and vortexed until the Coomassie brilliant blue was completely removed. To reduce the cysteine residues, each gel band was covered with a 10 mM DTT solution prepared in 50 mM (NH_4_)HCO_3_ for 60 min at 56 °C. The DTT solution was removed, and the excised bands were incubated with 55 mM iodoacetamide prepared in 50 mM (NH_4_)HCO_3_ for 40 min in the dark. The iodoacetamide solution was then removed. After washing the gel particles three times with 50 mM (NH_4_)HCO_3_ for 10 min, dehydration was performed with 100% acetonitrile for 10 min. The gel particles were then vacuum-dried for 20 min and rehydrated with 12.5 μg/μL trypsin (Promega) in 50 mM (NH_4_)HCO_3_ buffer. Digestion was performed by incubation at 37 °C overnight. Following digestion, tryptic peptides were extracted with 100 μl of 50% acetonitrile/1% acetic acid for 20 min. The tryptic peptides were dried with speed-vac, re-dissolved in 40 μl 0.1% acetic acid and subjected to LC-MS/MS analysis as described by Chen et al. (Chen, van der Schors et al. 2011). In short, 20 μL peptides were loaded on a 5 mm Pepmap 100 C18 (Dionex) column (300 μm ID, 5 μm particle size) and separated on a 200 mm Alltima C18 homemade column (100 μm ID, 3 μm particle size) with an Eksigent HPLC system, using a linear gradient of increasing acetonitrile concentration from 5 to 35% in 45 min, and to 90% in 5 min. The flow rate was 400 nL/min. The eluted peptides were electro-sprayed into the LTQ-Orbitrap discovery. The mass spectrometer was operated in a data dependent manner with one MS (m/z range from 330 to 2000) followed by MS-MS on five most abundant ions. The exclusion window was 25 s. MS/MS spectra were searched against an IPI Arabidopsis database (ipi.ARATH.v3.85) with the ProteinPilot software (version 3.0; Applied Biosystems, Foster City, CA, USA; MDS Sciex, https://sciex.com/products/software/proteinpilot-software) using the Paragon algorithm (version 3.0.0.0) as the search engine. The search parameters were set to cysteine alkylation with acrylamide, and digestion with trypsin. Detected protein threshold was set in protein summary to 0.5 achieving a 20% confidence. In this experiment, we included the unused values generated from the software ProteinPilot^31^. The ‘unused’ value is defined as a summation of protein scores from all the non-redundant peptides matched to a single protein. Peptides with confidence of > 99% have a protein score of 2; > 95% have a protein score of 1.3, > 66% have a protein score of 0.47, etc. The mass spectrometric data was searched against the Uniprot proteomics database (version 2013-01-06) with the Max-Quant software (version 1.3.0.5) to obtain peptides and proteins identified in each experiment. The search parameters were: MS accuracy 6 ppm, MS-MS accuracy 0.5 Da, fixed modification of cysteine alkyation with acrylamide, variable modification of methionine oxidation and protein N-terminal acetylation, digestion with trypsin, protein hits containing at least one unique peptide, and false discovery rates of both peptides and proteins within 0.01.

### Data analysis

To increase the data quality, we removed the low confident interacting proteins from the protein list. First, contaminant proteins like keratin and trypsin were removed from this results file. Second, considering the reproducibility of the pull-down experiment, we excluded proteins that were identified only once in all replicates. False positives were removed from the bait-prey matrix by comparing the abundance of proteins identified in each pull-down against their abundance in the matching empty bead controls, in which the true positive bait-prey interactions should be > tenfold enriched in the APs. Also, the four bait proteins were excluded from the list.

Intensity-based absolute quantification (iBAQ) values were calculated in the Max-Quant suite as previously described^63,64^. The protein abundance was calculated on the basis of the normalized iBAQ intensity. For short, quantifiable proteins in the analysis defined as those identified in at least two of the three biological replicates in at least one type of sample. Missing values were imputed using row mean imputation. The relative protein intensities were calculated as the ratio of their intensity to the bait proteins in that run. The relative protein intensities for each pull-down experiment were combined in a matrix, and false positives were removed the proteins identified in the background. The minimum two requirements for the differentially expressed proteins are: (1) identification of a protein with unused value > 2; (2) the fold change of protein quantities protein quantities in Fe-deficient treated samples against Fe-sufficient samples with more or less than 1.5 times with significant difference Student’s *t *test *P *value < 0.05). Functional analysis of identified proteins was obtained by performing Kyoto Encyclopedia of Genes and Genomes (KEGG) pathway analyses using the KEGG Orthology-Based Annotation System^65,66^.

## Supplementary Information

Supplementary Information.
