# Supplementary material for: Physiological and interactomic analysis reveals versatile functions of Arabidopsis 14-3-3 quadruple mutants in response to Fe deficiency
Source: Sci Rep. 2021 Jul 30;11:15551. doi: 10.1038/s41598-021-94908-9 (PMC8324900; doi:10.1038/s41598-021-94908-9)
Supplement: Supplementary file 1 — Supplementary Information. [file 41598_2021_94908_MOESM1_ESM.docx]

Supplementary Information

**Fig. S1.** Coomassie-stained SDS-PAGE gel of proteins isolated by 14-3-3 pull-down. CBB-stained gel was used as the loading control.

**Fig. S2.** KEGG pathway enrichment analysis of 14-3-3 potential target proteins.

**Table S1.** Proteins identified in pull-down assay with *At*14-3-3 coated beads and Wt root extracts (+ and –Fe).

**Table S2.** The KEGG pathway annotation of proteins identified in pull-down assay.

**Table S3** Interaction network analysis of identified proteins using the STRING software and database (<http://string.embl.de>), based on known and predicted interactions.

**Table S4.** 14-3-3 Interactome changes in Wt roots induced by Fe-deficiency.

**Table S5.** 14-3-3 Interactome changes in *klun* roots induced by Fe-deficiency.

**Table S6.** 14-3-3 Interactome differences between Wt and *klun* roots grown with sufficient Fe.

**Table S7.** The enriched KEGG pathway for changed 14-3-3 putative target protein in Wt.

**Table S8.** The enriched KEGG pathway for changed 14-3-3 putative target protein in *klun*.

**Table S9**. Primer pairs used for qRT-PCR according to Yang et al.

**A B**

**Background-empty beads**


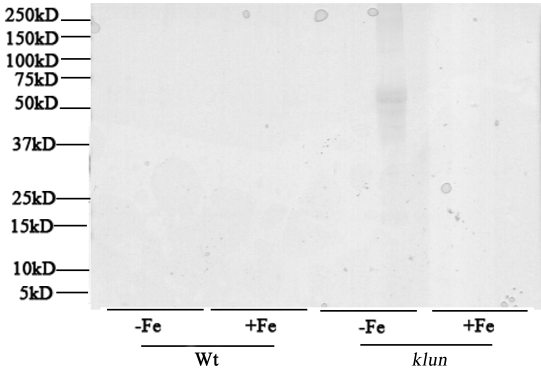


**Pull down-14-3-3 coated beads**

**14-3-3**

**protein mixture**


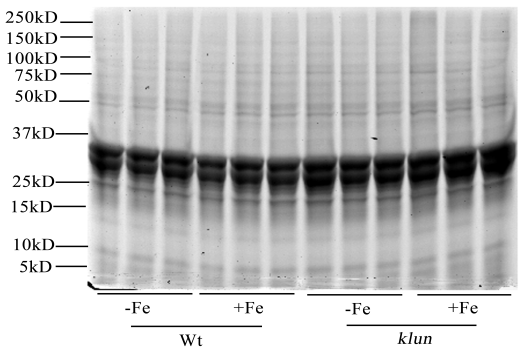


**Fig. S1. Coomassie-stained SDS-PAGE gel of proteins isolated by 14-3-3 pull-down.** **CBB-stained gel was used as the loading control. A.** Pull-down assay with empty beads and extracts of roots grown under Fe-deficient condition. **B.** Pull-down assay with At14-3-3 coated beads and extracts of roots grown under Fe-deficient condition. The numbers in the panel show the molecular weights of the protein marker of the ladder. For the pull-down assay nickel-beads were coated with N-terminally His-labelled 14-3-3 KAPPA, LAMBDA, NU and UPSILON in equimolar ratios (1:1:1:1:1). Roots were treated with or without 20 μM Fe-EDTA for 24 hours. Eluted fractions were separated on 10% SDS-PAGE.


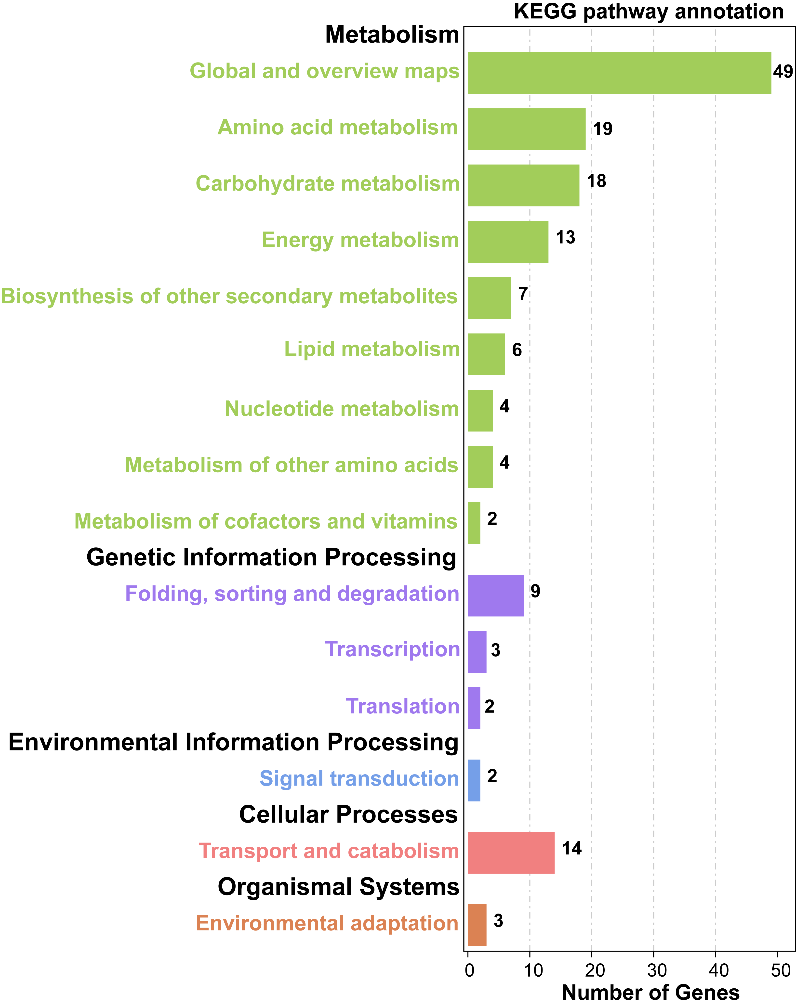


**Fig. S2. KEGG pathway enrichment analysis of 14-3-3 potential target proteins.** The left y-axis indicates the KEGG's A-level and B-level categories. The black font is the A-level category name, and the color font is the B-level category name; x-axis indicates the number of proteins in the corresponding B-level classification. See also Table S2 shown the whole KEGG enrichment analysis of the 117 identified proteins.

**Table S1. Proteins identified in pull-down assay with *At*14-3-3 coated beads and Wt root extracts (+ and –Fe).** Merging of all identified proteins in six independent pull-downs resulted in a list of 117 interacting proteins with unused value >2.

| **No.^a)^** | **ATG ^b)^** | **Protein name** | **Number of replicates ^c)^** | **Max unused ^d)^** | **Identified in previous 14-3-3 interactome studies ^e)^** | **protein changes in Fe deficiency proteomics study ^f)^** |
| --- | --- | --- | --- | --- | --- | --- |
| **23** | AT5G08670 | mitochondrial ion transporting ATP synthase beta-subunit | 6 | 24.62 | B,D |  |
| **24** | AT4G31340 | myosin heavy chain-related protein | 6 | 2.02 |  |  |
| **26** | AT1G79530 | GAPCP-1 (glyceraldehyde-3-phosphate dehydrogenase of plastid 1 | 6 | 6.38 |  |  |
| **34** | AT3G52930 | FBA8 (Fructose-bisphosphate aldolase 8) | 6 | 2.14 | B,C | G |
| **35** | AT1G27450 | APT1（Isoform 2 of Adenine phosphoribosyltransferase 1） | 4 | 6 |  |  |
| **36** | AT1G13440 | GAPC2 (glyceraldehyde-3-phosphate dehydrogenase c2) | 6 | 32.12 | B,C,D |  |
| **38** | AT4G35810  AT2G17720 | 2OG-Fe(II) oxygenase family protein | 2 | 2.04 |  |  |
| **39** | AT1G23490 | ARF1 (ADP-ribosylation factor 1) | 6 | 2 |  |  |
| **49** | AT1G48030 | mtLPD1 (Dihydrolipoyl dehydrogenase 1) | 2 | 2 |  | F |
| **56** | AT1G51470 | BGLU35 (Beta glucosidase 35) | 2 | 2.59 |  |  |
| **59** | AT5G08530 | CI51 (NADH dehydrogenase flavoprotein 1) | 6 | 2.82 |  |  |
| **79** | AT1G24280 | G6PD3 (Glucose-6-phosphate 1-dehydrogenase 3) | 6 | 9.22 | A | F (0.60) |
| **86** | AT1G09430 | ACLA-3 (ATP-citrate synthase alpha chain protein 3) | 4 | 2 |  |  |
| **88** | AT3G04120 | GAPC1 (Glyceraldehyde-3-phosphate dehydrogenase) | 6 | 6.38 | B,C,D |  |
| **94** | AT1G35580 | CINV1 (cytosolic invertase 1) | 6 | 46.07 | A,D | G |
| **96** | AT4G04040  AT1G12000 | MEE51 (maternal effect embryo arrest 51) | 6 | 2.18 |  |  |
| **108** | AT3G02090 | MPPBETA (Isoform 1 of Probable mitochondrial-processing peptidase subunit beta) | 6 | 10 |  |  |
| **110** | AT4G09510 | CINV2 (cytosolic invertase2) | 6 | 31.32 |  |  |
| **134** | AT5G13450 | ATP5 (ATP synthase subunit delta) | 6 | 10.92 | C |  |
| **151** | AT4G35260 | IDH1 (Isocitrate dehydrogenase regulatory subunit 1) | 6 | 2.43 |  |  |
| **159** | AT4G34860 | beta-fructofuranosidase invertase | 3 | 2.7 | D | G |
| **164** | AT1G63000 | UDP-4-keto-6-deoxy-glucose-3,5-epimerase | 3 | 3.4 |  |  |
| **170** | AT3G19820 | DWF1 (Delta(24)-sterol reductase) | 6 | 6 |  |  |
| **175** | ATMG01190  AT2G07698 | ATP1 (ATP synthase subunit alpha) | 6 | 7.53 |  |  |
| **42** | AT4G35790 | ATPLDDELTA (phospholipase D) | 2 | 2.21 |  |  |
| **47** | AT2G34470 | UREG (urease accessory protein g) | 6 | 5.4 |  | F |
| **61** | AT4G39980 | DHS1 (Phospho-2-dehydro-3-deoxyheptonate aldolase 1) | 6 | 6.59 | A |  |
| **65** | AT4G14360 | PMT3 (Probable methyltransferase 3) | 2 | 2.17 |  |  |
| **66** | AT5G17920 | ATMS1 (5-methyltetrahydropteroyltriglutamate--homocysteine methyltransferase) | 6 | 16.05 | B,D | G |
| **85** | AT1G52760 | LysoPL2 (lysophospholipase 2) | 6 | 2 |  |  |
| **91** | AT2G36880 | MAT3 (S-adenosylmethionine synthase 3) | 6 | 14.01 | C | E, F (1.47) |
| **93** | AT5G13640 | PDAT (Phospholipid:diacylglycerol acyltransferase 1) | 6 | 5.31 |  |  |
| **100** | AT4G01850 | MAT2 (S-adenosylmethionine synthase 2) | 2 | 2.45 |  | F (1.36) |
| **111** | AT5G43780 | APS4 (ATP sulfurylase 4, chloroplastic) | 6 | 6.01 |  |  |
| **115** | AT4G13930 | SHM4 (serine hydroxymethyltransferase 4) | 6 | 14 |  |  |
| **126** | AT1G22410 | 2-dehydro-3-deoxyphosphoheptonate aldolase | 6 | 10.1 |  |  |
| **129** | AT3G17390 | MAT 4 (S-adenosylmethionine synthase 4) | 6 | 9.98 |  |  |
| **146** | AT2G38040 | CAC3 (acetyl co-enzyme a carboxylase carboxyltransferase alpha subunit) | 4 | 2.39 |  |  |
| **147** | AT2G13360 | AGT (Serine-glyoxylate aminotransferase) | 6 | 2.89 |  |  |
| **149** | AT5G46290 | KAS I (Isoform 2 of 3-oxoacyl-[acyl-carrier-protein] synthase I) | 6 | 2.04 |  |  |
| **158** | AT4G13430 | IIL1 (3-isopropylmalate dehydratase) | 6 | 2.4 |  |  |
| **163** | AT1G02500 | MAT1 (S-adenosylmethionine synthase 1) | 6 | 3.51 |  | F (1.61) |
| **168** | AT5G54810 | TSB1 (Tryptophan synthase beta chain 1) | 4 | 2 |  |  |
| **25** | AT5G54430 | PHOS32 | 6 | 3.29 |  |  |
| **32** | AT5G45490 | Probable disease resistance protein | 6 | 4 |  |  |
| **33** | AT2G20990 | SYTA (Synaptotagmin A) | 3 | 2.6 |  |  |
| **50** | AT5G01600 | FER1 (Ferritin-1) | 6 | 2.85 |  | F (0.30) |
| **55** | AT4G35090 | CAT2 (catalase 2) | 6 | 6.3 | A |  |
| **60** | AT4G34050 | CCoAOMT1 (caffeoyl-CoA O-methyltransferase) | 6 | 8.59 |  | F (1.45) |
| **67** | AT5G64100 | Peroxidase 69 | 6 | 12.72 |  |  |
| **69** | AT5G38940 | Germin-like protein subfamily 1 member 11 | 5 | 14 |  |  |
| **133** | AT1G09560 | Germin-like protein subfamily 1 member 5, GLP5 | 6 | 14 |  | E ,F (4.50) |
| **82** | AT5G45510 | Isoform 2 of Probable disease resistance protein | 2 | 4.01 |  |  |
| **99** | AT3G15950 | NAI2 | 6 | 14.95 |  |  |
| **119** | AT4G24800 | MA3 domain-containing protein | 4 | 4.01 |  |  |
| **139** | AT5G14030 | TRAPB (translocon-associated protein beta) | 3 | 2 |  |  |
| **142** | AT4G23690 | AtDIR6 (Arabidopsis thaliana dirigent protein 6) | 3 | 2 |  |  |
| **145** | AT4G21960 | PRXR1 (Peroxidase 42) | 6 | 5.25 |  |  |
| **162** | AT4G27320 | PHOS34 | 6 | 6.3 |  |  |
| **173** | AT1G49570 | Peroxidase 10 | 3 | 4.12 |  |  |
| **176** | AT1G72330 | ALAAT2 (Alanine aminotransferase 2) | 5 | 2.01 |  | F |
| **31** | AT1G56070 | LOS1 (Elongation factor EF-2) | 6 | 6.06 | B,D |  |
| **54** | AT4G20360 | RABFAFFEOYL | 2 | 4.27 |  |  |
|  |  | E1b (Elongation factor Tu) |  |  |  |  |
| **63** | AT3G13920 | EIF4A1 (eukaryotic translation initiation factor 4A1) | 6 | 8.01 | A,B |  |
| **64** | AT1G63660 | GMP synthase (glutamine amidotransferase) | 6 | 22.27 |  |  |
| **95** | AT4G02930 | Elongation factor Tu | 6 | 10.7 |  |  |
| **153** | AT1G26630 | FBR12 (Eukaryotic translation initiation factor 5A-2) | 6 | 9.77 | B |  |
| **172** | AT1G07920 | GTP binding Elongation factor Tu family protein | 6 | 29.58 | B,D |  |
| **41** | AT3G15980 | Isoform 1 of Coatomer subunit beta-3 | 3 | 3.3 |  |  |
| **62** | AT3G11830 | TCP-1/cpn60 chaperonin family protein, eta subunit | 6 | 11.19 |  |  |
| **102** | AT3G03960 | TCP-1/cpn60 chaperonin family protein, theta subunit | 2 | 2 |  |  |
| **103** | AT3G18190 | TCP-1/cpn60 chaperonin family protein, delta subunit | 5 | 3 |  |  |
| **112** | AT5G26360 | TCP-1/cpn60 chaperonin family protein, gamma subunit | 2 | 2 |  |  |
| **117** | AT1G67760 | TCP-1/cpn60 chaperonin family protein, epsilon subunit | 6 | 2.64 |  |  |
| **118** | AT3G53110 | LOS4 (low expression of osmotically responsive genes 4) | 4 | 2.13 |  |  |
| **121** | AT5G56030 | HSP81-2 (Heat shock protein 81-2) | 6 | 4.85 | A,B,D |  |
| **122** | AT5G20890 | TCP-1/cpn60 chaperonin family protein | 6 | 2.83 |  |  |
| **140** | AT2G47470 | UNE5 (unfertilized embryo sac 5) | 2 | 2 |  |  |
| **141** | AT5G61790 | CNX1 (Calnexin homolog 1) | 6 | 4.31 |  |  |
| **156** | AT5G02500 | HSC70-1 (heat shock cognate protein 70-1) | 6 | 6.01 | B,C |  |
| **166** | AT3G08580 | AAC1 (ATP carrier protein 1) | 6 | 6.76 |  |  |
| **37** | AT1G30630 | Coatomer subunit epsilon-1 | 6 | 4 |  |  |
| **70** | AT1G76030 | V-type proton ATPase subunit B1 | 3 | 2.04 |  |  |
| **81** | AT1G78900 | VHA-A (V-type proton ATPase catalytic subunit A) | 2 | 4 |  |  |
| **127** | AT4G32285 | Probable clathrin assembly protein | 2 | 2 |  |  |
| **144** | AT4G11150 | TUF (V-type proton ATPase subunit E1) | 3 | 2 |  |  |
| **27** | AT1G75780 | TUB1 (Tubulin beta-1) | 6 | 14.93 |  |  |
| **53** | AT4G14960 | TUA6 (Tubulin/FtsZ family protein) | 6 | 16.37 | A |  |
| **72** | AT3G18780 | ACT2 (Actin-2) | 6 | 12 |  |  |
| **73** | AT5G09810 | ACT7 (Actin-7) | 6 | 9.22 | A,B |  |
| **92** | AT1G11580 | PMEPCRA (/pectinesterase inhibitor 18) | 6 | 4 | B |  |
| **98** | AT5G19770 | TUA3 (Tubulin alpha-3 chain) | 6 | 4.14 |  | G |
| **152** | AT5G44340 | TUB4 (Tubulin beta-4 chain) | 6 | 14.93 |  |  |
| **167** | AT5G55480 | SVL1 (Probable glycerophosphoryl diester phosphodiesterase 1) | 2 | 2 |  |  |
| **169** | AT1G04820 | TUA2 (Tubulin alpha-2) | 6 | 18.73 | B | G |
| **40** | AT1G56330 | GTP-binding protein (Fragment) | 3 | 2 | B |  |
| **52** | AT3G52990 | pyruvate kinase family protein | 6 | 2.02 |  |  |
|  |  |  |  |  |  |  |
| **75** | AT4G35310 | CPK5 (Calcium-dependent protein kinase 5) | 4 | 3.41 |  |  |
| **89** | AT1G14000 | VIK F16A14.22 | 6 | 3.85 |  |  |
| **97** | AT4G23650 | CDPK6 (Calcium-dependent protein kinase 3) | 6 | 14.04 |  |  |
| **105** | AT2G20900 | DGK5 (diacylglycerol kinase 5) | 5 | 2 |  |  |
| **113** | AT3G63260 | ATMRK1 (protein serine/threonine/tyrosine kinase) | 2 | 2.06 |  |  |
| **114** | AT3G52400 | SYP122 (Syntaxin-122) | 6 | 6 |  |  |
| **120** | AT4G18950 | ankyrin protein kinase, putative | 6 | 6.95 |  |  |
| **148** | AT5G63190 | MA3 domain-containing protein | 4 | 2 |  |  |
| **43** | AT4G29830 | VIP3 (vernalization independence 3) | 6 | 2 |  |  |
| **74** | AT2G14120 | DRP3B (dynamin-like protein 3b) | 6 | 5.1 |  |  |
| **78** | AT5G44500 | small nuclear ribonucleoprotein associated protein B | 2 | 2.06 |  |  |
| **106** | AT4G32720 | La1 AtLa1 (Arabidopsis thaliana La protein 1) | 6 | 18.06 |  |  |
| **123** | AT1G09760 | U2A U2 small nuclear ribonucleoprotein A | 6 | 2.04 |  |  |
| **132** | AT4G38680 | GRP2, CSP2 (Cold shock protein 2 | 3 | 2 |  |  |
| **138** | AT5G23540 | 26S proteasome regulatory subunit, putative | 6 | 2.02 | B |  |
| **157** | AT2G24420 | DNA repair ATPase-related protein | 2 | 4.01 |  |  |
| **174** | AT4G38220 | N-acyl-L-amino-acid amidohydrolase, putative | 6 | 4.21 |  |  |
| **109** | AT1G58270 | ZW9 | 6 | 3.64 |  |  |
| **51** | AT5G26290 | meprin and TRAF homology domain-containing protein | 3 | 2 |  |  |
| **116** | AT4G09000 | GRF1 (14-3-3-like protein GF14 chi) | 3 | 2.01 |  |  |

a) Order of identified protein in the protein list searched against the Uniprot proteomics database.

b) ATG number: the accession number of Arabidopsis genes.

c) Number of replicates: how often a protein was identified among different replicates.

d) Max unused: the maximum unused value among different replicates.

e) Identified in previous 14-3-3 interactome study: proteins that have been reported in previous 14-3-3 interactome studies. A=Chang *et al*, 2009; B=Paul *et al*,2009; C=Shin *et al.*,2011; D=Swatek *et al.*, 2011.

f) Proteins whose abundance changes during Fe deficiency (F; Lan *et al.*, 2011 and López-Millán *et al*., 2013; in brackets the fold-change) and proteins that show enhanced phosphorylation under Fe-deficiency conditions (G = Lan *et al*., 2012).

**Table S2. The KEGG pathway annotation of proteins identified in pull-down assay.**

| **KEGG_A_class** | **KEGG_B_class** | **Pathway** | **out (117)** | **All (4748)** | **Qvalue** | **Pathway ID** | **Genes** |  |
| --- | --- | --- | --- | --- | --- | --- | --- | --- |
| Metabolism | Global and overview maps | Biosynthesis of amino acids | 18 | 247 | 7.95E-07 | ko01230 | AT1G02500; | AT3G52930 |
|  |  |  |  |  |  |  | AT1G13440; | AT3G52990 |
|  |  |  |  |  |  |  | AT1G22410; | AT4G01850 |
|  |  |  |  |  |  |  | AT1G72330; | AT4G13430 |
|  |  |  |  |  |  |  | AT1G79530; | AT4G13930 |
|  |  |  |  |  |  |  | AT2G36880; | AT4G35260 |
|  |  |  |  |  |  |  | AT3G04120; | AT4G38220 |
|  |  |  |  |  |  |  | AT3G17390; | AT4G39980 |
|  |  |  |  |  |  |  | AT5G54810; | AT5G17920 |
| Cellular Processes | Transport and catabolism | Phagosome | 9 | 82 | 6.33E-05 | ko04145 | AT1G04820; | AT4G14960 |
|  |  |  |  |  |  |  | AT1G75780; | AT5G19770 |
|  |  |  |  |  |  |  | AT1G76030; | AT5G44340 |
|  |  |  |  |  |  |  | AT1G78900; | AT5G61790 |
|  |  |  |  |  |  |  | AT4G11150; |  |
| Metabolism | Global and overview maps | Metabolic pathways | 48 | 1893 | 6.33E-05 | ko01100 | AT1G02500; | AT3G19820 |
|  |  |  |  |  |  |  | AT1G09430; | AT3G52930 |
|  |  |  |  |  |  |  | AT1G11580; | AT3G52990 |
|  |  |  |  |  |  |  | AT1G12000; | AT4G01850 |
|  |  |  |  |  |  |  | AT1G13440; | AT4G04040 |
|  |  |  |  |  |  |  | AT1G22410; | AT4G11150 |
|  |  |  |  |  |  |  | AT1G24280; | AT4G13430 |
|  |  |  |  |  |  |  | AT1G27450; | AT4G13930 |
|  |  |  |  |  |  |  | AT1G48030; | AT4G21960 |
|  |  |  |  |  |  |  | AT1G49570; | AT4G34050 |
|  |  |  |  |  |  |  | AT1G52760; | AT4G35260 |
|  |  |  |  |  |  |  | AT1G63660; | AT4G35790 |
|  |  |  |  |  |  |  | AT1G72330; | AT4G35810 |
|  |  |  |  |  |  |  | AT1G76030; | AT4G38220 |
|  |  |  |  |  |  |  | AT1G78900; | AT4G39980 |
|  |  |  |  |  |  |  | AT1G79530; | AT5G08530 |
|  |  |  |  |  |  |  | AT2G07698; | AT5G08670 |
|  |  |  |  |  |  |  | AT2G13360; | AT5G13450 |
|  |  |  |  |  |  |  | AT2G17720; | AT5G13640 |
|  |  |  |  |  |  |  | AT2G20900; | AT5G17920 |
|  |  |  |  |  |  |  | AT2G36880; | AT5G43780 |
|  |  |  |  |  |  |  | AT2G38040; | AT5G46290 |
|  |  |  |  |  |  |  | AT3G04120; | AT5G54810 |
|  |  |  |  |  |  |  | AT3G17390; | AT5G64100 |
| Metabolism | Global and overview maps | Biosynthesis of secondary metabolites | 33 | 1075 | 1.43E-04 | ko01110 | AT1G02500; | AT3G52930 |
|  |  |  |  |  |  |  | AT1G09430; | AT3G52990 |
|  |  |  |  |  |  |  | AT1G12000; | AT4G01850 |
|  |  |  |  |  |  |  | AT1G13440; | AT4G04040 |
|  |  |  |  |  |  |  | AT1G22410; | AT4G13430 |
|  |  |  |  |  |  |  | AT1G24280; | AT4G13930 |
|  |  |  |  |  |  |  | AT1G48030; | AT4G21960 |
|  |  |  |  |  |  |  | AT1G49570; | AT4G34050 |
|  |  |  |  |  |  |  | AT1G52760; | AT4G35090 |
|  |  |  |  |  |  |  | AT1G79530; | AT4G35260 |
|  |  |  |  |  |  |  | AT2G13360; | AT4G35790 |
|  |  |  |  |  |  |  | AT2G20900; | AT4G38220 |
|  |  |  |  |  |  |  | AT2G36880; | AT4G39980 |
|  |  |  |  |  |  |  | AT2G38040; | AT5G17920 |
|  |  |  |  |  |  |  | AT3G04120; | AT5G54810 |
|  |  |  |  |  |  |  | AT3G17390; | AT5G64100 |
|  |  |  |  |  |  |  | AT3G19820; |  |
| Metabolism | Global and overview maps | Carbon metabolism | 13 | 260 | 1.30E-03 | ko01200 | AT1G13440; | AT3G04120 |
|  |  |  |  |  |  |  | AT1G24280; | AT3G52930 |
|  |  |  |  |  |  |  | AT1G48030; | AT3G52990 |
|  |  |  |  |  |  |  | AT1G72330; | AT4G13930 |
|  |  |  |  |  |  |  | AT1G79530; | AT4G35090 |
|  |  |  |  |  |  |  | AT2G13360; | AT4G35260 |
|  |  |  |  |  |  |  | AT2G38040; |  |
| Metabolism | Carbohydrate metabolism | Glycolysis / Gluconeogenesis | 8 | 116 | 3.07E-03 | ko00010 | AT1G12000; | AT3G04120 |
|  |  |  |  |  |  |  | AT1G13440; | AT3G52930 |
|  |  |  |  |  |  |  | AT1G48030; | AT3G52990 |
|  |  |  |  |  |  |  | AT1G79530; | AT4G04040 |
| Metabolism | Energy metabolism | Oxidative phosphorylation | 7 | 126 | 2.25E-02 | ko00190 | AT1G76030; | AT5G08530 |
|  |  |  |  |  |  |  | AT1G78900; | AT5G08670 |
|  |  |  |  |  |  |  | AT2G07698; | AT5G13450 |
|  |  |  |  |  |  |  | AT4G11150; |  |
| Metabolism | Energy metabolism | Carbon fixation in photosynthetic organisms | 5 | 68 | 2.47E-02 | ko00710 | AT1G13440; | AT3G04120 |
|  |  |  |  |  |  |  | AT1G72330; | AT3G52930 |
|  |  |  |  |  |  |  | AT1G79530; |  |
| Metabolism | Carbohydrate metabolism | Pentose phosphate pathway | 4 | 58 | 7.05E-02 | ko00030 | AT1G12000; | AT3G52930 |
|  |  |  |  |  |  |  | AT1G24280; | AT4G04040 |
| Metabolism | Amino acid metabolism | Glycine, serine and threonine metabolism | 4 | 70 | 1.20E-01 | ko00260 | AT1G48030; | AT4G13930 |
|  |  |  |  |  |  |  | AT2G13360; | AT5G54810 |
| Metabolism | Global and overview maps | 2-Oxocarboxylic acid metabolism | 4 | 74 | 1.21E-01 | ko01210 | AT1G72330; | AT4G35260 |
|  |  |  |  |  |  |  | AT4G13430; | AT4G38220 |
| Metabolism | Carbohydrate metabolism | Glyoxylate and dicarboxylate metabolism | 4 | 77 | 1.21E-01 | ko00630 | AT1G48030; | AT4G13930 |
|  |  |  |  |  |  |  | AT2G13360; | AT4G35090 |
| Metabolism | Amino acid metabolism | Cysteine and methionine metabolism | 5 | 115 | 1.21E-01 | ko00270 | AT1G02500; | AT4G01850 |
|  |  |  |  |  |  |  | AT2G36880; | AT5G17920 |
|  |  |  |  |  |  |  | AT3G17390; |  |
| Metabolism | Metabolism of other amino acids | Selenocompound metabolism | 2 | 18 | 1.21E-01 | ko00450 | AT5G17920; | AT5G43780 |
| Metabolism | Amino acid metabolism | Phenylalanine, tyrosine and tryptophan biosynthesis | 3 | 56 | 1.99E-01 | ko00400 | AT1G22410; | AT5G54810 |
|  |  |  |  |  |  |  | AT4G39980; |  |
| Metabolism | Carbohydrate metabolism | Citrate cycle (TCA cycle) | 3 | 63 | 2.42E-01 | ko00020 | AT1G09430; | AT4G35260 |
|  |  |  |  |  |  |  | AT1G48030; |  |
| Metabolism | Carbohydrate metabolism | Fructose and mannose metabolism | 3 | 64 | 2.42E-01 | ko00051 | AT1G12000; | AT4G04040 |
|  |  |  |  |  |  |  | AT3G52930; |  |
| Metabolism | Carbohydrate metabolism | Propanoate metabolism | 2 | 32 | 2.66E-01 | ko00640 | AT1G48030; | AT2G38040 |
| Genetic Information Processing | Folding, sorting and degradation | Protein processing in endoplasmic reticulum | 6 | 211 | 2.66E-01 | ko04141 | AT1G56330; | AT5G14030 |
|  |  |  |  |  |  |  | AT2G47470; | AT5G56030 |
|  |  |  |  |  |  |  | AT5G02500; | AT5G61790 |
| Metabolism | Carbohydrate metabolism | C5-Branched dibasic acid metabolism | 1 | 7 | 2.66E-01 | ko00660 | AT4G13430; |  |
| Metabolism | Biosynthesis of other secondary metabolites | Stilbenoid, diarylheptanoid and gingerol biosynthesis | 1 | 7 | 2.66E-01 | ko00945 | AT4G34050; |  |
| Metabolism | Amino acid metabolism | Arginine biosynthesis | 2 | 36 | 2.66E-01 | ko00220 | AT1G72330; | AT4G38220 |
| Metabolism | Biosynthesis of other secondary metabolites | Phenylpropanoid biosynthesis | 5 | 168 | 2.73E-01 | ko00940 | AT1G49570; | AT4G34050 |
|  |  |  |  |  |  |  | AT1G52760; | AT5G64100 |
|  |  |  |  |  |  |  | AT4G21960; |  |
| Metabolism | Lipid metabolism | Fatty acid biosynthesis | 2 | 40 | 2.91E-01 | ko00061 | AT2G38040; | AT5G46290 |
| Metabolism | Carbohydrate metabolism | Pyruvate metabolism | 3 | 85 | 3.14E-01 | ko00620 | AT1G48030; | AT3G52990 |
|  |  |  |  |  |  |  | AT2G38040; |  |
| Metabolism | Amino acid metabolism | Alanine, aspartate and glutamate metabolism | 2 | 49 | 3.72E-01 | ko00250 | AT1G72330; | AT2G13360 |
| Metabolism | Biosynthesis of other secondary metabolites | Monobactam biosynthesis | 1 | 14 | 3.87E-01 | ko00261 | AT5G43780; |  |
| Metabolism | Amino acid metabolism | Arginine and proline metabolism | 2 | 54 | 3.87E-01 | ko00330 | AT2G17720; | AT4G35810 |
| Metabolism | Amino acid metabolism | Tryptophan metabolism | 2 | 54 | 3.87E-01 | ko00380 | AT1G51470; | AT4G35090 |
| Metabolism | Nucleotide metabolism | Purine metabolism | 4 | 157 | 3.99E-01 | ko00230 | AT1G27450; | AT3G52990 |
|  |  |  |  |  |  |  | AT1G63660; | AT5G43780 |
| Metabolism | Metabolism of cofactors and vitamins | Biotin metabolism | 1 | 16 | 3.99E-01 | ko00780 | AT5G46290; |  |
| Metabolism | Lipid metabolism | Glycerolipid metabolism | 2 | 59 | 0.401 | ko00561 | AT2G20900; | AT5G13640 |
| Metabolism | Metabolism of cofactors and vitamins | One carbon pool by folate | 1 | 20 | 4.43E-01 | ko00670 | AT4G13930; |  |
| Metabolism | Global and overview maps | Fatty acid metabolism | 2 | 66 | 4.43E-01 | ko01212 | AT2G38040; | AT5G46290 |
| Metabolism | Amino acid metabolism | Valine, leucine and isoleucine biosynthesis | 1 | 22 | 4.53E-01 | ko00290 | AT4G13430; |  |
| Metabolism | Biosynthesis of other secondary metabolites | Flavonoid biosynthesis | 1 | 22 | 4.53E-01 | ko00941 | AT4G34050; |  |
| Metabolism | Biosynthesis of other secondary metabolites | Glucosinolate biosynthesis | 1 | 23 | 4.57E-01 | ko00966 | AT4G13430; |  |
| Metabolism | Lipid metabolism | Ether lipid metabolism | 1 | 27 | 5.08E-01 | ko00565 | AT4G35790; |  |
| Cellular Processes | Transport and catabolism | Endocytosis | 3 | 144 | 5.44E-01 | ko04144 | AT1G23490; | AT5G02500 |
|  |  |  |  |  |  |  | AT4G35790; |  |
| Cellular Processes | Transport and catabolism | Peroxisome | 2 | 87 | 5.44E-01 | ko04146 | AT2G13360; | AT4G35090 |
| Metabolism | Lipid metabolism | Steroid biosynthesis | 1 | 34 | 5.62E-01 | ko00100 | AT3G19820; |  |
| Metabolism | Lipid metabolism | Glycerophospholipid metabolism | 2 | 93 | 5.62E-01 | ko00564 | AT2G20900; | AT4G35790 |
| Metabolism | Energy metabolism | Sulfur metabolism | 1 | 38 | 5.86E-01 | ko00920 | AT5G43780; |  |
| Organismal Systems | Environmental adaptation | Plant-pathogen interaction | 3 | 170 | 6.08E-01 | ko04626 | AT4G23650; | AT5G56030 |
|  |  |  |  |  |  |  | AT4G35310; |  |
| Metabolism | Amino acid metabolism | Phenylalanine metabolism | 1 | 43 | 6.08E-01 | ko00360 | AT4G34050; |  |
| Genetic Information Processing | Folding, sorting and degradation | SNARE interactions in vesicular transport | 1 | 44 | 6.08E-01 | ko04130 | AT3G52400; |  |
| Metabolism | Amino acid metabolism | Valine, leucine and isoleucine degradation | 1 | 47 | 6.23E-01 | ko00280 | AT1G48030; |  |
| Genetic Information Processing | Transcription | Spliceosome | 3 | 192 | 6.68E-01 | ko03040 | AT1G09760; | AT5G44500 |
|  |  |  |  |  |  |  | AT5G02500; |  |
| Genetic Information Processing | Folding, sorting and degradation | Proteasome | 1 | 60 | 7.01E-01 | ko03050 | AT5G23540; |  |
| Metabolism | Metabolism of other amino acids | Cyanoamino acid metabolism | 1 | 67 | 7.33E-01 | ko00460 | AT4G13930; |  |
| Metabolism | Carbohydrate metabolism | Pentose and glucuronate interconversions | 1 | 76 | 7.56E-01 | ko00040 | AT1G11580; |  |
| Environmental Information Processing | Signal transduction | Phosphatidylinositol signaling system | 1 | 76 | 7.56E-01 | ko04070 | AT2G20900; |  |
| Genetic Information Processing | Translation | RNA transport | 2 | 171 | 7.95E-01 | ko03013 | AT1G07920; | AT3G13920 |
| Metabolism | Metabolism of other amino acids | Glutathione metabolism | 1 | 102 | 8.37E-01 | ko00480 | AT1G24280; |  |
| Genetic Information Processing | Folding, sorting and degradation | RNA degradation | 1 | 113 | 8.56E-01 | ko03018 | AT4G29830; |  |
| Metabolism | Carbohydrate metabolism | Amino sugar and nucleotide sugar metabolism | 1 | 131 | 8.69E-01 | ko00520 | AT1G63000; |  |
| Environmental Information Processing | Signal transduction | MAPK signaling pathway - plant | 1 | 131 | 8.69E-01 | ko04016 | AT4G35090; |  |

**Table S3 Interaction network analysis of identified proteins using the STRING software and database** (<http://string.embl.de>), **based on known and predicted interactions.** Lines of different colors represent different types of evidence for the associations. Three major protein complexes were identified: mFoF1-synthase, V-ATPase, Tubulin. Four functional networks were identified: elongation factors, chaperones, glycolysis and TCA cycle and cysteine/methionine metabolism.

| **Categories** | **ATG** | **Network** |
| --- | --- | --- |
| mFoF1-synthase  complex | AT3G02090  AT5G13450  AT5G08670  ATMG01190  AT2G07698  AT5G08530 | 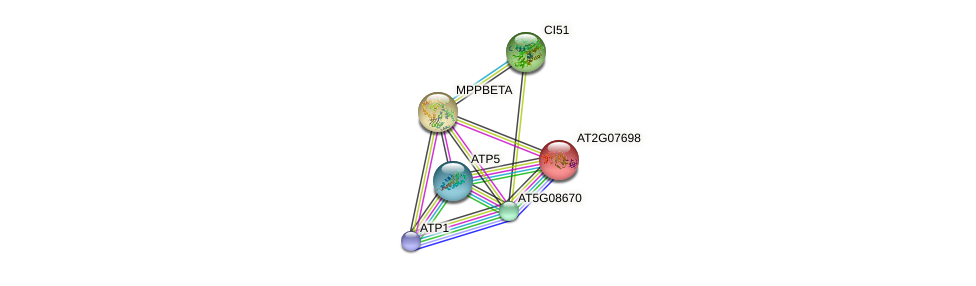 |
| V-ATPase complex | AT1G76030  AT4G11150  AT1G78900 | 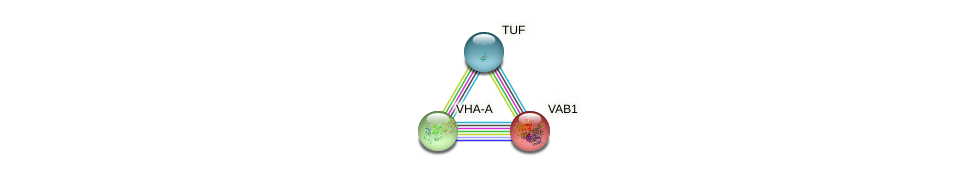 |
| Tubulin complex | AT1G75780  AT4G14960  AT3G18780  AT5G09810  AT5G19770  AT5G44340  AT1G04820 | 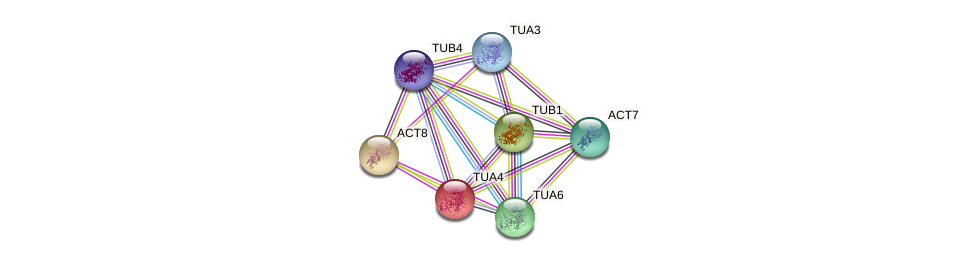 |
| Elongation factor network | AT1G07920  AT4G20360  AT3G13920  AT4G02930  AT1G26630  AT1G56070 | 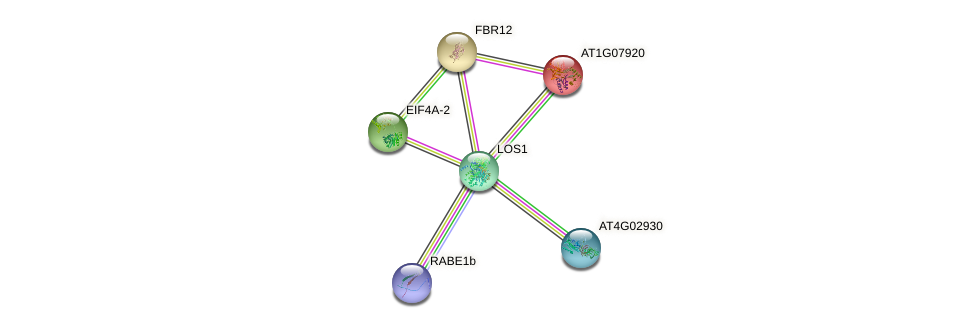 |
| Chaperone network | AT5G56030  AT5G20890  AT2G47470  AT5G61790  AT5G02500  AT3G11830  AT3G03960  AT3G18190  AT5G26360  AT1G67760 | 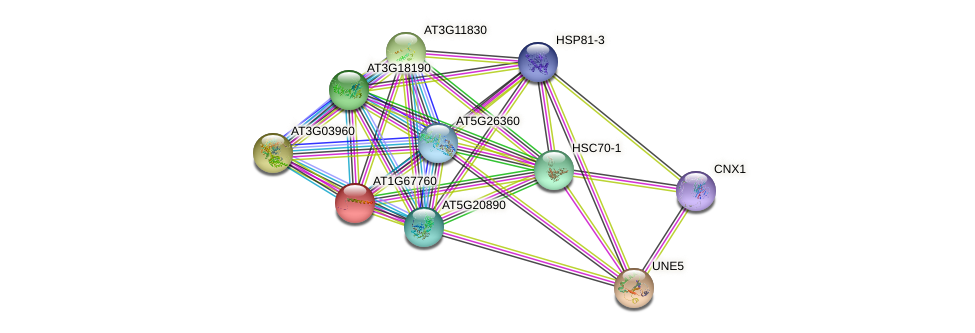 |
| Glycolysis and TCA cycle | AT1G79530  AT3G52930  AT1G13440  AT1G48030  AT1G24280  AT1G09430  AT3G04120  AT4G04040  AT4G35260 | 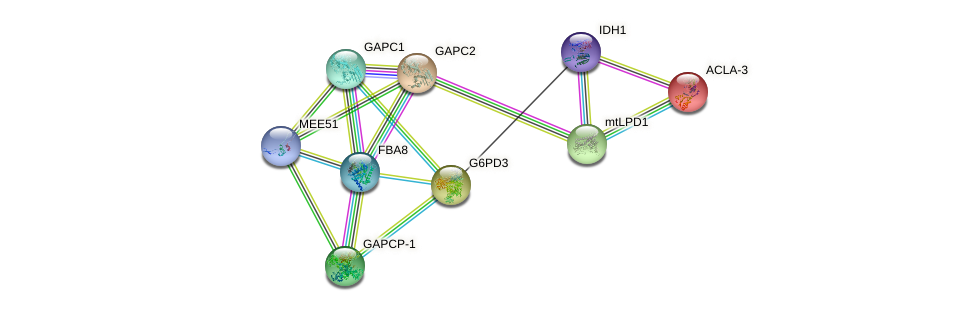 |
| Cysteine and methionine metabolism network | AT5G17920  AT2G36880  AT4G01850  AT4G13930  AT3G17390  AT1G02500 | 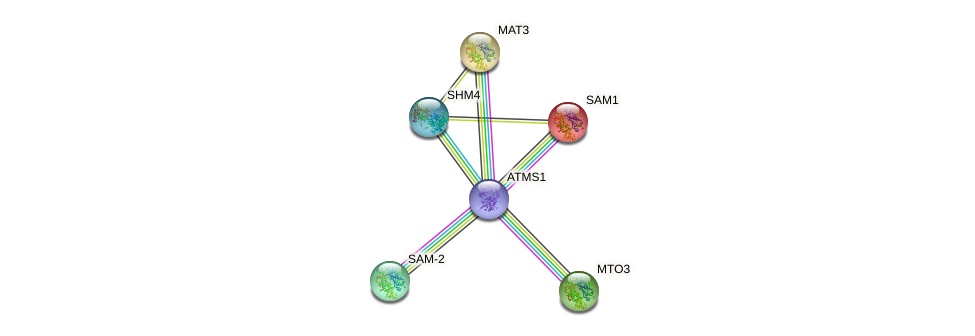 |

**Table S4. 14-3-3 Interactome changes in Wt roots induced by Fe-deficiency.** Interacting proteins were classified as Fe responsive when the ratio -Fe/+Fe was higher than 1.5 (increase in binding) or lower than 0.66 (decrease in binding) with a 95% confidence level (*P<0.05*, Students’s t-test). Ratios were calculated from three biological replicates. Interactors are ranked according to functional categories. A ratio of zero implies that a protein found in the +Fe treatment was lost in the -Fe treatment.

| **Num. ^a)^** | **ATG number ^b)^** | | **Name** | | **Fold**  **-Fe/+Fe** | ***p* value** |
| --- | --- | --- | --- | --- | --- | --- |
| **Up-regulated** | | | |  |  |  |
| **108** | | AT3G02090 | | MPPBETA (mitochondrial-processing peptidase subunit beta) | 10.20 | 0.036 |
| **134** | | AT5G13450 | | ATP5 (ATP synthase subunit delta) | 4.4 | 0.015 |
| **34** | | AT3G52930 | | FBA8 (fructose-bisphosphate aldolase) | 4.03 | 0.008 |
| **59** | | AT5G08530 | | CI51 (NADH dehydrogenase flavoprotein 1) | 3.2 | 2E^-04^ |
| **175** | | ATMG01190 | | ATP1 (ATP synthase subunit alpha) | 2.44 | 0.013 |
| **26** | | AT1G79530 | | GAPCP-1 (glyceraldehyde-3- phosphate dehydrogenase) | 2 | 0.041 |
| **23** | | AT5G08670 | | mitochondrial ion transporting ATP synthase beta-subunit | 1.82 | 0.034 |
| **163** | | AT1G02500 | | MAT1 (S-adenosylmethionine synthase 1) | 3.36 | 0.004 |
| **117** | | AT1G67760 | | TCP-1/cpn60 chaperonin family protein | 2.91 | 0.035 |
| **31** | | AT1G56070 | | LOS1 (Elongation factor EF-2) | 2.57 | 0.05 |
| **43** | | AT4G29830 | | VIP3 (vernalization independence 3) | 1.74 | 0.031 |
| **25** | | AT5G54430 | | PHOS32 | 2.12 | 0.009 |
| **142** | | AT4G23690 | | Dirigent protein 6 | N.A.^c)^ | 0.008 |
| **Down-regulated** | | | |  |  |  |
| **56** | | AT1G51470 | | BGLU35, TGG5  (Beta glucosidase 35) | 0 ^c)^ | 0.002 |
| **79** | | AT1G24280 | | G6PD3 (\Glucose-6-phosphate 1-dehydrogenase 3) | 0.31 | 0.012 |
| **110** | | AT4G09510 | | CINV2 (Neutral invertase 2) | 0.53 | 0.021 |
| **129** | | AT3G17390 | | MAT4 (S-adenosylmethionine synthase 4) | 0.26 | 0.01 |
| **66** | | AT5G17920 | | ATMS1 (5-methyltetrahydropteroyl triglutamate-homocysteine methyltransferase) | 0.48 | 0.006 |
| **62** | | AT3G11830 | | TCP-1/cpn60 chaperonin family protein, eta subunit | 0.31 | 0.042 |
| **33** | | AT2G20990 | | SYTA (Synaptotagmin A) | 0 | 0.027 |
| **145** | | AT4G21960 | | PRXR1 (Peroxidase 42) | 0.65 | 0.041 |
| **119** | | AT4G24800 | | ECIP1 ( EIN2 C-terminus interacting protein 1) | 0 | 0.016 |
| **105** | | AT2G20900 | | DGK5 (diacylglycerol kinase), putative | 0 | 0.001 |
| **116** | | AT4G09000 | | GRF1 (14-3-3-like protein GF14 chi) | 0 | 5E^-04^ |
| **97** | | AT4G23650 | | CDPK6 (Calcium-dependent protein kinase 3) | 0.38 | 0.023 |
| **120** | | AT4G18950 | | ankyrin protein kinase, putative | 0.40 | 0.021 |
| **109** | | AT1G58270 | | ZW9 | 0 | 0.001 |

a) Protein number identified is listed in Table S1.

b) ATG number: the accession number of Arabidopsis genes.

c) 0 means that the protein was not found in –Fe roots, NA means that the protein was not found in the +Fe roots.

**Table S5. 14-3-3 Interactome changes in *klun* roots induced by Fe-deficiency.** Proteins with a ratio >1.5 are more abundant in the pull-down from Wt roots as compared to *klun*, whereas proteins with a ratio <0.66 are more abundant in the pull-down from *klun* roots as compared to Wt. Ratios were calculated from three biological replicates and only proteins with significant ratio difference are shown (*P<0.05*, Student’s *t*-test).

| **Num.^a)^** | | **ATG number ^b)^** | | **Name** | **Fold** | ***p* value** |
| --- | --- | --- | --- | --- | --- | --- |
| **Up-regulated** | | |  | |  |  |
| 163 | AT1G02500 | | MAT1 (S-adenosylmethionine synthase 1) | | 4.05 | 0.005 |
| 175 | ATMG01190 | | ATP1 (ATP synthase subunit alpha, mitochondrial) | | 2.1 | 0.0004 |
| 55 | AT4G35090 | | CAT2 (CATALASE 2); catalase | | 1.9 | 0.012 |
| 34 | AT3G52930 | | fructose-bisphosphate aldolase, putative | | 1.86 | 0.059 |
| 31 | AT1G56070 | | LOS1 (Elongation factor EF-2) | | 1.77 | 0.016 |
| 74 | AT2G14120 | | DRP3B (dynamin-like protein 2b) | | 1.73 | 0.021 |
| 66 | AT5G17920 | | ATMS1 (5-methyltetrahydropteroyltriglutamate-homocysteine methyltransferase) | | 1.57 | 0.023 |
| **Down-regulated** | | |  | |  |  |
| 91 | AT2G36880 | | MAT3 (S-adenosylmethionine synthase 3) | | 0.64 | 0.025 |
| 67 | AT5G64100 | | Peroxidase 69 | | 0.61 | 0.041 |
| 138 | AT5G23540 | | 26S proteasome regulatory subunit, putative | | 0.57 | 0.025 |
| 151 | AT4G35260 | | IDH1 Isocitrate dehydrogenase [NAD] regulatory subunit 1, mitochondrial | | 0.41 | 0.043 |
| 106 | AT4G32720 | | La1 AtLa1 (Arabidopsis thaliana La protein 1); RNA binding | | 0.36 | 0.045 |
| 111 | AT5G43780 | | APS4 ATP sulfurylase 4, chloroplastic | | 0.35 | 0.003 |
| 129 | AT3G17390 | | MAT4 (S-adenosylmethionine synthase 4) | | 0.29 | 0.012 |
| 61 | AT4G39980 | | DHS1 Phospho-2-dehydro-3-deoxyheptonate aldolase 1, chloroplastic | | 0.29 | 0.03 |
| 120 | AT4G18950 | | ankyrin protein kinase, putative | | 0.2 | 0.003 |
| 162 | AT4G27320 | | PHOS34 universal stress protein (USP) family protein | | 0.15 | 0.003 |

a) Protein number identified is listed in Table S1.

b) ATG number: the accession number of Arabidopsis genes.

c) 0 means that the protein was not found in Wt roots, NA means that the protein was not found in the *klun* roots

**Table S6. 14-3-3 Interactome differences between Wt and *klun* roots grown with sufficient Fe.** Proteins with a ratio >1.5 are more abundant in the pull-down from Wt roots as compared to *klun*, whereas proteins with a ratio <0.66 are more abundant in the pull-down from *klun* roots as compared to Wt. Ratios were calculated from three biological replicates and only proteins with significant ratio difference are shown (*P<0.05*, Student’s *t*-test).

| **Num. ^a)^** | **ATG number ^b)^** | **Name** | **Wt / *klun*** | |
| --- | --- | --- | --- | --- |
|  |  |  | **Fold** | ***p* value** |
| **Up-regulated** | |  |  |  |
| 175 | AT2G07698 | ATP1 (ATP synthase subunit alpha) | 2.46 | 0.049 |
| 134 | AT5G13450 | ATP5 (ATP synthase subunit delta) | 2.46 | 0.050 |
| 56 | AT1G51470 | BGLU35 (Beta glucosidase 35) | N.A. ^c)^ | 0.001 |
| 110 | AT4G09510 | CINV2 (cytosolic invertase 2) | N.A.. | 0.009 |
| 43 | AT4G29830 | VIP3 (vernalization independence 3) | 1.55 | 0.044 |
| 123 | AT1G09760 | U2A (U2 small nuclear ribonucleoprotein A) | N.A. | 0.052 |
| 69 | AT5G38940 | Germin-like protein subfamily 1 member 11 | N.A. | 0.005 |
| 173 | AT1G49570 | Peroxidase 10 | N.A. | 0.002 |
| 142 | AT4G23690 | Dirigent protein 6 | N.A. | 0.040 |
| 148 | AT5G63190 | MA3 domain-containing protein | N.A. | 0.002 |
| **Down-regulated** | |  |  |  |
| 35 | AT1G27450 | APT1 (Isoform 2 of Adenine phosphoribosyltransferase 1) | 0.26 | 0.000 |
| 36 | AT1G13440 | GAPC2 (glyceraldehyde-3-phosphate dehydrogenase C2) | 0.30 | 0.027 |
| 88 | AT3G04120 | GAPC1 (Glyceraldehyde-3-phosphate dehydrogenase) | 0.33 | 0.013 |
| 94 | AT1G35580 | CINV1 (cytosolic invertase 1) | 0.44 | 0.020 |
| 147 | AT2G13360 | AGT (Serine-glyoxylate aminotransferase) | 0.18 | 0.009 |
| 149 | AT5G46290 | KAS I (Isoform 2 of 3-oxoacyl-[acyl-carrier  -protein] synthase I) | 0.25 | 0.035 |
| 146 | AT2G38040 | CAC3 (Acetyl-coenzyme A carboxylase carboxyl transferase subunit alpha) | 0.31 | 0.004 |
| 41 | AT3G15980 | Coatomer subunit beta'-3 | 0.00^c^ | 0.016 |
| 176 | AT1G72330 | ALAAT2 (Alanine aminotransferase 2) | 0.00 | 0.030 |
| 133 | AT3G05950 | Germin-like protein subfamily 1 member 7 | 0.29 | 0.026 |
| 60 | AT4G34050 | CCoAOMT1 (Probable caffeoyl-CoA O-methyltransferase) | 0.30 | 0.013 |
| 162 | AT4G27320 | PHOS34 | 0.43 | 0.013 |
| 132 | AT4G38680 | GRP2, CSP2 (Cold shock protein 2) | 0.00 | 0.011 |
| 95 | AT4G02930 | Elongation factor Tu | 0.16 | 0.030 |
| 172 | AT1G07920 | GTP binding Elongation factor Tu family protein | 0.34 | 0.001 |
| 27 | AT1G75780 | TUB1 Tubulin beta-1 chain TUB5 Tubulin beta-5 | 0.18 | 0.012 |
| 53 | AT4G14960 | TUA6 (Tubulin/FtsZ family protein) | 0.25 | 0.002 |
| 72 | AT3G18780 | ACT2 (Actin-2) | 0.28 | 0.048 |
| 73 | AT5G09810 | ACT7 (Actin-7) | 0.28 | 0.035 |
| 152 | AT5G44340 | TUB4 (Tubulin beta-4 chain) | 0.29 | 0.025 |
| 70 | AT1G76030 | V-type proton ATPase subunit B1 | 0.00 | 0.005 |
| 116 | AT4G09000 | GRF1 (14-3-3-like protein GF14 chi) | 0.10 | 0.003 |

a) Protein number identified is listed in Table S1.

b) ATG number: the accession number of Arabidopsis genes.

c) 0 means that the protein was not found in Wt roots, NA means that the protein was not found in the *klun* roots

**Table S7. The enriched KEGG pathway for changed 14-3-3 putative target protein in Wt.**

| **KEGG_A_class** | **KEGG_B_class** | **Pathway** | **out (14)** | **All (4748)** | **Qvalue** | **Pathway ID** | **Genes** |
| --- | --- | --- | --- | --- | --- | --- | --- |
| Metabolism | Global and overview maps | Biosynthesis of amino acids | 5 | 247 | 0.009448 | ko01230 | AT1G02500;AT1G79530;  AT3G17390;AT3G52930;AT5G17920 |
| Metabolism | Global and overview maps | Metabolic pathways | 11 | 1893 | 0.020466 | ko01100 | AT1G02500;AT1G24280;  AT1G79530;AT2G20900;AT3G17390;  AT3G52930;AT4G21960;AT5G08530;  AT5G08670;AT5G13450;AT5G17920 |
| Metabolism | Amino acid metabolism | Cysteine and methionine metabolism | 3 | 115 | 0.020466 | ko00270 | AT1G02500;AT3G17390;AT5G17920 |
| Metabolism | Energy metabolism | Oxidative phosphorylation | 3 | 126 | 0.020466 | ko00190 | AT5G08530;AT5G08670;AT5G13450 |
| Metabolism | Global and overview maps | Biosynthesis of secondary metabolites | 8 | 1075 | 0.020466 | ko01110 | AT1G02500;AT1G24280;AT1G79530;  AT2G20900;AT3G17390;AT3G52930;  AT4G21960;AT5G17920 |
| Metabolism | Carbohydrate metabolism | Pentose phosphate pathway | 2 | 58 | 0.038464 | ko00030 | AT1G24280;AT3G52930 |
| Metabolism | Energy metabolism | Carbon fixation in photosynthetic organisms | 2 | 68 | 0.044677 | ko00710 | AT1G79530;AT3G52930 |
| Metabolism | Global and overview maps | Carbon metabolism | 3 | 260 | 0.089549 | ko01200 | AT1G24280;AT1G79530;AT3G52930 |
| Metabolism | Carbohydrate metabolism | Glycolysis / Gluconeogenesis | 2 | 116 | 0.093872 | ko00010 | AT1G79530;AT3G52930 |
| Metabolism | Metabolism of other amino acids | Selenocompound metabolism | 1 | 18 | 0.098526 | ko00450 | AT5G17920 |
| Metabolism | Amino acid metabolism | Tryptophan metabolism | 1 | 54 | 0.253216 | ko00380 | AT1G51470 |
| Metabolism | Lipid metabolism | Glycerolipid metabolism | 1 | 59 | 0.253216 | ko00561 | AT2G20900 |
| Metabolism | Carbohydrate metabolism | Fructose and mannose metabolism | 1 | 64 | 0.253216 | ko00051 | AT3G52930 |
| Environmental Information Processing | Signal transduction | Phosphatidylinositol signaling system | 1 | 76 | 0.274763 | ko04070 | AT2G20900 |
| Metabolism | Lipid metabolism | Glycerophospholipid metabolism | 1 | 93 | 0.306777 | ko00564 | AT2G20900 |
| Metabolism | Metabolism of other amino acids | Glutathione metabolism | 1 | 102 | 0.311691 | ko00480 | AT1G24280 |
| Genetic Information Processing | Folding, sorting and degradation | RNA degradation | 1 | 113 | 0.320299 | ko03018 | AT4G29830 |
| Metabolism | Biosynthesis of other secondary metabolites | Phenylpropanoid biosynthesis | 1 | 168 | 0.400207 | ko00940 | AT4G21960 |
| Organismal Systems | Environmental adaptation | Plant-pathogen interaction | 1 | 170 | 0.400207 | ko04626 | AT4G23650 |

**Table S8. The enriched KEGG pathway for changed 14-3-3 putative target protein in *klun*.**

| **KEGG_A_class** | **KEGG_B_class** | **Pathway** | **out (11)** | **All (4748)** | **Qvalue** | **Pathway ID** | **Genes** |
| --- | --- | --- | --- | --- | --- | --- | --- |
| Metabolism | Global and overview maps | Biosynthesis of amino acids | 7 | 247 | 5.76E-06 | ko01230 | AT1G02500;AT2G36880;AT3G17390;AT3G52930;AT4G35260;AT4G39980;AT5G17920 |
| Metabolism | Global and overview maps | Biosynthesis of secondary metabolites | 9 | 1075 | 5.87E-04 | ko01110 | AT1G02500;AT2G36880;AT3G17390;AT3G52930;AT4G35090;AT4G35260;AT4G39980;AT5G17920;AT5G64100 |
| Metabolism | Amino acid metabolism | Cysteine and methionine metabolism | 4 | 115 | 6.93E-04 | ko00270 | AT1G02500;AT2G36880;AT3G17390;AT5G17920 |
| Metabolism | Metabolism of other amino acids | Selenocompound metabolism | 2 | 18 | 4.02E-03 | ko00450 | AT5G17920;AT5G43780 |
| Metabolism | Global and overview maps | Metabolic pathways | 9 | 1893 | 2.52E-02 | ko01100 | AT1G02500;AT2G36880;AT3G17390;AT3G52930;AT4G35260;AT4G39980;AT5G17920;AT5G43780;AT5G64100 |
| Metabolism | Global and overview maps | Carbon metabolism | 3 | 260 | 7.07E-02 | ko01200 | AT3G52930;AT4G35090;AT4G35260 |
| Metabolism | Biosynthesis of other secondary metabolites | Monobactam biosynthesis | 1 | 14 | 1.01E-01 | ko00261 | AT5G43780 |
| Metabolism | Energy metabolism | Sulfur metabolism | 1 | 38 | 2.13E-01 | ko00920 | AT5G43780 |
| Metabolism | Amino acid metabolism | Tryptophan metabolism | 1 | 54 | 2.13E-01 | ko00380 | AT4G35090 |
| Metabolism | Amino acid metabolism | Phenylalanine, tyrosine and tryptophan biosynthesis | 1 | 56 | 2.13E-01 | ko00400 | AT4G39980 |
| Metabolism | Carbohydrate metabolism | Pentose phosphate pathway | 1 | 58 | 2.13E-01 | ko00030 | AT3G52930 |
| Genetic Information Processing | Folding, sorting and degradation | Proteasome | 1 | 60 | 2.13E-01 | ko03050 | AT5G23540 |
| Metabolism | Carbohydrate metabolism | Citrate cycle (TCA cycle) | 1 | 63 | 2.13E-01 | ko00020 | AT4G35260 |
| Metabolism | Carbohydrate metabolism | Fructose and mannose metabolism | 1 | 64 | 2.13E-01 | ko00051 | AT3G52930 |
| Metabolism | Energy metabolism | Carbon fixation in photosynthetic organisms | 1 | 68 | 2.13E-01 | ko00710 | AT3G52930 |
| Metabolism | Global and overview maps | 2-Oxocarboxylic acid metabolism | 1 | 74 | 2.13E-01 | ko01210 | AT4G35260 |
| Metabolism | Carbohydrate metabolism | Glyoxylate and dicarboxylate metabolism | 1 | 77 | 2.13E-01 | ko00630 | AT4G35090 |
| Cellular Processes | Transport and catabolism | Peroxisome | 1 | 87 | 2.25E-01 | ko04146 | AT4G35090 |
| Metabolism | Carbohydrate metabolism | Glycolysis / Gluconeogenesis | 1 | 116 | 2.76E-01 | ko00010 | AT3G52930 |
| Environmental Information Processing | Signal transduction | MAPK signaling pathway - plant | 1 | 131 | 2.92E-01 | ko04016 | AT4G35090 |
| Metabolism | Nucleotide metabolism | Purine metabolism | 1 | 157 | 3.24E-01 | ko00230 | AT5G43780 |
| Metabolism | Biosynthesis of other secondary metabolites | Phenylpropanoid biosynthesis | 1 | 168 | 3.27E-01 | ko00940 | AT5G64100 |

**Table S9.** **Primer pairs used for qRT-PCR according to Yang et al ^4.^**

| Gene | Locus | Primers |
| --- | --- | --- |
| *IRT1* | AtU27590 | Fw:5'-GCGACTTGTAGTGCGGCTATG-3' |
|  |  | Rev:5'-CGTTGCACGAGCGATTCTG-3' |
| *FRO2* | At1g01580 | Fw:5'-GGAGAAGGTGTTGCTCCATCTC-3' |
|  |  | Rev:5'-GGTTAGGCAAGTTTAAGCTCTG-3' |
| *AHA2* | At4g30190 | Fw:5'-TTTGCCGGAGTCTTCCCAGGT-3' |
|  |  | Rev:5'-GGGGCATCATTGACACCATCACCAG-3' |
| *FIT* | At2g28160 | Fw:5'-GTATCAATCCTCCTGCTT-3' |
|  |  | Rev:5'-TCTCGGTTACATCATCACT-3' |
| *14-3-3 omicron* | At1g34760 | Fw:5'-CTATCACTCAAGGCGTATG-3' |
|  |  | Rev:5'-GTATGGCTCCGTAACTAAG-3' |
| *UBQ10* | At4g05320 | Fw:5'- GTCTTCGTGGTGGTTTCTAAATCT -3' |
|  |  | Rev:5'- TTATTCATCAGGGATTATACAAGG -3' |
|  |  |  |
